# Selective targeting of parallel G-quadruplex structure using L-RNA aptamer

**DOI:** 10.1093/nar/gkad900

**Published:** 2023-10-23

**Authors:** Danyang Ji, Jia-Hao Yuan, Shuo-Bin Chen, Jia-Heng Tan, Chun Kit Kwok

**Affiliations:** Department of Chemistry and State Key Laboratory of Marine Pollution, City University of Hong Kong, Kowloon Tong, Hong Kong SAR, China; Guangdong Provincial Key Laboratory of New Drug Design and Evaluation, School of Pharmaceutical Sciences, Sun Yat-sen University, Guangzhou 510006, China; Guangdong Provincial Key Laboratory of New Drug Design and Evaluation, School of Pharmaceutical Sciences, Sun Yat-sen University, Guangzhou 510006, China; Guangdong Provincial Key Laboratory of New Drug Design and Evaluation, School of Pharmaceutical Sciences, Sun Yat-sen University, Guangzhou 510006, China; Department of Chemistry and State Key Laboratory of Marine Pollution, City University of Hong Kong, Kowloon Tong, Hong Kong SAR, China; Shenzhen Research Institute of City University of Hong Kong, Shenzhen, China

## Abstract

G-quadruplexes (G4) are special nucleic acid structures with diverse conformational polymorphisms. Selective targeting of G-quadruplex conformations and regulating their biological functions provide promising therapeutic intervention. Despite the large repertoire of G4-binding tools, only a limited number of them can specifically target a particular G4 conformation. Here, we introduce a novel method, G4-SELEX-Seq and report the development of the first L-RNA aptamer, L-Apt12-6, with high binding selectivity to parallel G4 over other nucleic acid structures. Using parallel dG4 c-*kit* 1 as an example, we demonstrate the strong binding affinity between L-Apt12-6 and c-*kit* 1 dG4 *in vitro* and in cells, and notably report the applications of L-Apt12-6 in controlling DNA replication and gene expression. Our results suggest that L-Apt12-6 is a valuable tool for targeting parallel G-quadruplex conformation and regulating G4-mediated biological processes. Furthermore, G4-SELEX-Seq can be used as a general platform for G4-targeting L-RNA aptamers selection and should be applicable to other nucleic acid structures.

## Graphical Abstract

**Graphical Abstract:**
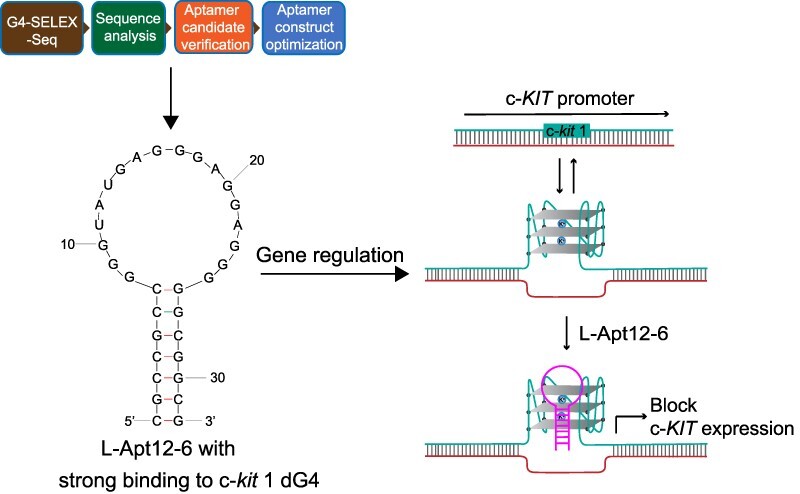

## Introduction

Nucleic acids can fold into diverse secondary structures, which are crucial for the function and regulation of diverse biological processes (1). Among them, a non-canonical four-stranded structure, named G-quadruplex (G4), is of increasing interest (2). G4s are found in guanine (G)-rich DNA and RNA sequences, formed by π-π stacking of two or more planar Hoogsteen hydrogen-bonded G-tetrads, and further stabilized by monovalent cations (2), such as K^+^ and Na^+^. Genome-wide and transcriptome-wide studies have shown that G4s are enriched in human telomeres, promoters of oncogenes and different regions of mRNA (3,4). Numerous studies have reported that G4s play critical roles in various cellular processes, including DNA replication, transcriptional and translational regulation, genome stability, maintenance of telomeres and RNA metabolism, and are involved in many diseases, such as cancers, making them potential targets for drug development and cancer therapeutics (5–7).

G4s possess rich conformational polymorphisms, which can be described as parallel, antiparallel and hybrid conformation based on the orientation of the strands (2). Conformational information on G4 is important for *in vitro* applications, such as G4-based biosensor design, and in cellular studies to understand its biological functions. Development and application of synthetic molecules to target functional G4s and regulate G4-mediated processes can provide insights into biological mechanisms and promising therapeutic intervention (8,9). However, it is challenging to distinguish G4 conformation *in vitro* and in cells because of its structural complexity, which depends on many parameters, including DNA/RNA sequences and environmental factors, such as cations, molecular crowding and ligand binding (10). Traditional G4-targeting tools, including fluorescent probes (9), small-molecule ligands (8) and antibodies (11) have been developed. Despite their high selectivity to G4 compared to other structural motifs, such as single strands, duplexes and hairpins, only a few of them can target specific G4 conformation (12–15), while most ligands lack G4 conformational selectivity, and thus are unable to discriminate among different G4 conformations. Therefore, it is important to develop a new platform and generate novel tools that can specifically interact with specific G4 conformation.

**Figure 1.**
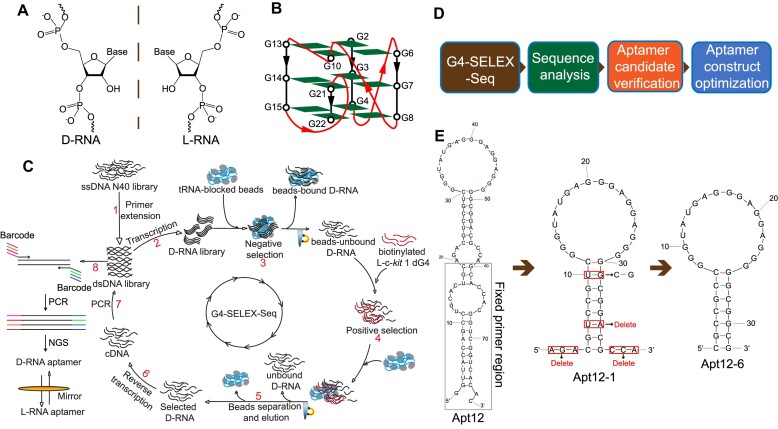
Selection of L-RNA aptamer for c-*kit* 1 dG4 by G4-SELEX-Seq. (**A**) Chemical structures of D-RNA and L-RNA. (**B**) Schematic representation of the c-*kit* 1 parallel G-quadruplex structure. (**C**) Flowchart of G4-SELEX-Seq. D-RNA aptamers binding to the enantiomeric form of c-*kit* 1 dG4 target (L-c-*kit* 1 dG4) were first identified by *in vitro* selection. When the final D-RNA aptamer sequence was confirmed, L-RNA aptamer was then chemically synthesized to recognize D-c-*kit* 1 dG4 target. (**D**) Downstream processes after G4-SELEX-Seq to get an optimized aptamer. (**E**) Sequences and mFold predicted structures of full-length and truncated aptamers. The fixed primer region of full-length aptamer (Apt12) for G4-SELEX-Seq is boxed in gray. The N40 region (Apt12-1) was truncated by deleting the two flanking ends (5′-AGA and 3′-CCA) and the ‘U-A’ base pair in stem. To strengthen the stem region, the ‘U-G’ base pair originally in the stem region of Apt12-1 was mutated to ‘C-G’, and resulted in the final 32-nt aptamer sequence Apt12-6.

Aptamers are single-stranded nucleic acid sequences with specific secondary and tertiary structures that can specifically bind to their corresponding targets (16). Referred as chemical antibodies, aptamer possess excellent properties compared to traditional protein-based antibodies, such as simple synthesis and modification, smaller physical size and structure flexibility, making them attractive in biochemical and biological research (17). To select aptamers for DNA/RNA sequences by recognizing their tertiary structure rather than Watson-Crick pairing, unnatural aptamers comprising L-RNA have been developed (18–21). L-RNA is the enantiomeric form of natural D-RNA (Figure 1A), which cannot hybridize with D-DNA/RNA through contiguous Watson-Crick base-pairing, allowing cross-chiral recognition of natural DNA/RNA targets. L-RNA aptamers show strong stability under biological conditions owing to its resistance to nuclease degradation, making it well suited for biological applications (22,23). Motivated by these findings, we aim to develop an L-RNA aptamer for a specific G4 conformation.

In this study, we first refined the *in vitro* Systematic Evolution of Ligands by Exponential Enrichment (SELEX) strategy for L-RNA aptamers selection and developed an innovative selection platform, referred to as G4-SELEX-Seq. Using G4-SELEX-Seq with parallel dG4 c-*kit* 1 as the target (Figure 1B), we successfully identified and optimized an L-RNA aptamer, named L-Apt12-6, which can specifically bind to parallel G4, but not antiparallel or hybrid G4, with nanomolar affinity. We then investigated the applications of L-Apt12-6 to inhibit primer extension, regulate reporter gene expression and suppress endogenous c-*KIT* gene expression, indicating that L-Apt12-6 can selectively bind to parallel G4 and regulate gene activity.

## Materials and methods

### Preparation of oligonucleotides

All the L-RNA oligos used in this work (Supplementary Table S1) were ordered from ChemGenes Corporation (USA). All the D-RNA oligos (Supplementary Table S1) were ordered form IDT (USA). All the D-DNA oligos (Supplementary Table S1) were ordered from Genewiz Biotechnology Co., Ltd (Suzhou, China). The L-RNA/D-DNA/D-RNA powder was dissolved using nuclease-free distilled water and stocked at -30°C.

### *In vitro* selection (G4-SELEX-Seq)

The ssDNA library containing a random N40 region (Supplementary Table S1) was obtained from IDT (USA). The dsDNA library was obtained by primer extension on a 2 μM ssDNA library with 300 nM reverse primer (Supplementary Table S1), 10 U/μl Superscript III reverse transcriptase (Invitrogen, USA) and 1 mM dNTP mixture (Invitrogen, USA) in Tris–HCl buffer (20 mM, pH 7.5) with 4 mM MgCl_2_, 1 mM dithiothreitol (DTT) and 150 mM LiCl. The generated dsDNA products were purified by Zymo-Spin IC DNA Columns (Zymo Research, USA) and transcribed to an RNA library with a HiScribe T7 High Yield RNA Synthesis Kit (NEB, USA). The 40 μl *in vitro* transcription reaction mixture was incubated at 37°C for 2.5 h. The DNA templates were then degraded by adding 2 U Turbo DNase (Invitrogen, USA) and incubated at 37°C for another 15 min. The transcribed RNA library was purified by 10% denaturing polyacrylamide gel electrophoresis (PAGE) and subsequent Zymo-Spin IC RNA Column (Zymo Research, USA) purification.

To prepare for selection, MyOne™ Streptavidin C1 dynabeads (3 mg) (Thermo Fisher Scientific, USA) were washed and blocked with 0.1 mg/ml yeast tRNA (Thermo Fisher Scientific, USA) by shaking at 300 rpm and 25°C for 1 h. Specific amounts of RNA library (Supplementary Table S2) in selection buffer with 150 mM KCl, 1 or 5 mM MgCl_2_ (Supplementary Table S2), and 25 mM Tris–HCl was denatured at 75°C for 3 min and cooled down to room temperature (RT). Then, 1 mg of tRNA-blocked dynabeads was added to the RNA library for negative selection. The mixture was then incubated at 300 rpm and 25°C for 1 or 2 h (Supplementary Table S2). Beads were discarded to remove bead-bound RNAs. The supernatant was collected for positive selection with certain concentration of biotinylated L-c-*kit* 1 (Supplementary Table S2). The mixture was incubated at 25°C or 37°C for 30 or 60 min (Supplementary Table S2). Then, 2 mg of tRNA-blocked dynabeads were added and shaken at 25°C for 30 min. The supernatant was discarded after bead separation. Beads were washed five times using 600 μl of selection buffer before elution with 25 mM NaOH and 1 mM EDTA. The RNA solution obtained was neutralized by Tris–HCl buffer (pH 7.5), followed by RNA column purification. The purified RNAs were reverse-transcribed to cDNA with 300 nM reverse primer (Supplementary Table S1), 10 U/μl Superscript III reverse transcriptase, and 1 mM dNTP mixture in 60 μl Tris–HCl reaction buffer (20 mM, pH 7.5) with 4 mM MgCl_2_, 1mM DTT and 150 mM LiCl. After incubating at 50°C for 30 min, 3 μl of 2 M NaOH was added and heated at 95 ^o^C for 10 min to degrade RNA and protein. The DNA solution obtained was neutralized by 15 μl of 1M Tris–HCl (pH 7.5), followed by column purification. Then PCR reaction was carried out using cDNA, Q5 DNA polymerase master mix (NEB, USA), and 500 nM forward and reverse primers in a 40 μl reaction. After dsDNA column purification, the dsDNA library was used as a transcription template for the next round of selection. A total of four selection rounds were performed for Biotin-L-c-*kit* 1, and the conditions of each round, including RNA library input, MgCl_2_ concentration, negative selection time, L-c-*kit* 1 input, positive selection time and temperature, bead washing time and PCR cycles, are listed in Supplementary Table S2.

After four rounds of selection, the ssDNA library got from each round was amplified by PCR reaction to add barcode and linker sequences for next-generation sequencing (NGS) purpose (Supplementary Table S1). NGS was performed by Guangzhou IGE Biotechnology LTD (China). The procedures of TA cloning and plasmid extraction for Sanger Sequencing were the same as described previously by us (21).

### Electrophoretic mobility shift assay (EMSA)

Before the binding reaction, Apt12-6 (or L-Apt12-6) was annealed by heating at 95°C for 3 min, and then cooled to RT. Different concentrations of Apt12-6 (or L-Apt12-6) (0–2000 nM) were added to FAM-labelled L-c-*kit* 1 dG4 (or FAM-labelled D-c-*kit* 1 dG4) (10 nM) in 25 mM Tris–HCl buffer (pH 7.5) with 150 mM KCl and 10 mM MgCl_2_. After 1 h of incubation at 37°C, the binding results were analyzed using 10% non-denaturing PAGE (19:1, acrylamide: bis-acrylamide). The PAGE gel was prepared with running buffer containing 25 mM Tris–HCl (pH 7.5), 50 mM KOAc and 1 mM MgCl_2_. After running at 4°C with a consistent current of 70 mA for 40 min, gel images were collected by FLA-9000 Gel Scanner (FujiFilm) with a voltage of 650 V. The intensities of the bound (*I*_Bound_) and unbound bands (*I*_Unbound_) in EMSA gel images were quantified using ImageJ. Fraction Bound was calculated as *I*_Bound_/(*I*_Bound_ + *I*_Unbound_). Then the binding curve was fitted using the model of ‘Binding-Saturation-One Site-Specific binding’ in GraphPad Prism. The dissociation constant (*K*_d_) value was calculated by the software automatically based on the binding curve.

### Microscale thermophoresis (MST) assay

Before the binding reaction, L-Apt12-6 was annealed by heating at 95°C for 3 min, and then cooled to RT. Different concentrations of L-Apt12-6 (0–2000 nM) were added to FAM-labelled D-c-*kit* 1 dG4 (100 nM) in the binding buffer, the same as that used in EMSA. After 1 h's binding reaction at 37°C, the samples were transferred to a capillary tube for MST tests. The ‘nano-blue’ channel was selected on NanoTemper Monolith NT.115 instrument. Data were analyzed at *K*_d_ model in the NanoTemper Analysis software.

### Fluorescence detection of Apt12-6 with ThT/NMM

D/L-Apt12-6 (500 nM) was heated in binding buffer at 95°C for 3 min and cooled to RT. Then, ThT (Solarbio Life Science, China) or NMM (Frontier Specialty Chemicals, USA) (2 μM) was mixed with the D/L-Apt12-6 solution for 30 min at RT. After that, the solutions were transferred to a quartz cuvette with a path length of 1 cm for fluorescence testing using the HORIBA FluoroMax-4 fluorescence spectrophotometer from Tokyo, Japan. ThT and NMM were excited at 425 and 399 nm, respectively, and their respective emission wavelengths were recorded from 450 to 600 nm and 550 to 650 nm.

### Circular dichroism (CD) spectroscopy

D/L-Apt12-6 was subjected to heat treatment at 95°C for 3 min in binding buffer, followed by cooling to RT. Subsequently, the D/L-Apt12-6 solutions were analyzed for CD using a Jasco CD J-150 spectrometer equipped with a 1-cm path length quartz cuvette. Spectra from 220 to 320 nm were scanned at 1 nm intervals. Three scans for each sample were accumulated and averaged. For CD melting assay, the melting curve was monitored from 20°C to 95°C at 1.0°C intervals.

### UV spectroscopy

Following the preparation of the samples for CD analysis, samples for UV assays were prepared and tested. The UV spectra were scanned from 220 nm - 340 nm at 20°C and 95°C, separately. The UV melting curve at 295 or 260 nm was monitored from 20°C to 95°C at 0.5°C intervals using a Cary UV-Vis spectrophotometer.

### Dideoxy sequencing and reverse transcriptase stalling (RTS) assay

For the reverse transcriptase stalling assay, 500 nM of Apt12-6ext (Supplementary Table S1), 500 nM of 5′Cy5-labelled reverse SELEX primer, and 1 mM dNTP mix were added to 20 mM Tris–HCl buffer (pH 7.5, 4 mM MgCl_2_, 1 mM DTT and 150 mM LiCl/KCl). The mixture was heated at 75°C for 3 min and incubated at 35°C for 10 min before adding 100 U Superscript III reverse transcriptase. After a 15 min reaction at 50°C, 1 μl of 2 M NaOH was added and heated at 95°C for 10 min to degrade RNAs and proteins. For dideoxy sequencing, Apt12-6ext (Supplementary Table S1) (500 nM) was mixed with 500 nM of 5′Cy5 labelled reverse primer, 1 mM dNTP mix and 1 mM of corresponding dideoxynucleotides (ddATP, ddGTP, ddCTP, ddTTP) in Li^+^-containing reverse transcription buffer. The reaction conditions and procedures were the same as those used for the reverse transcriptase stalling assay. Denaturing PAGE (8%) was used to analyze the reaction products. The gel was performed at 80 W for 2 h and scanned by FujiFilm FLA-9000 Gel Imager.

### Selective 2′hydroxyl acylation analyzed lithium ion-mediated primer extension (SHALiPE)

A 20 μl binding reaction with 500 nM Apt12-6ext and different concentrations of L-c-*kit* 1 or L-SL1 RNA (0, 1, 5 and 10 μM) in 25 mM Tris–HCl buffer (pH 7.5, 50 mM KCl and 10 mM MgCl_2_) was prepared first. After 1 h of binding reaction at 37°C, 1 μl of 2 M NAI was added and incubated at 37°C for another 5 min to modify the RNA. Then, 5 μl of 2 M DTT was used to stop the reaction. After RNA column purification, acylated RNA was directly used for lithium ion-mediated reverse transcription. The following reaction procedures were the same as those used for the reverse transcriptase stalling assay described above.

### DNA polymerase stop assay

Different concentrations of L-Apt12-6 or BRACO-19 (0, 0.5, 1, 2, 5, 8 and 10 μM) were mixed with 100 nM wildtype/mutant dG4 containing PSA templates (Supplementary Table S1) and 100 nM FAM-labelled primers (Supplementary Table S1) in binding buffer. After 1 h of incubation at 37°C, DreamTaq DNA polymerase (1 U) (Thermo Fisher Scientific, USA) and 1 mM dNTP mix were added and incubated at 45°C for 30 min. The reaction products were loaded onto a 15% denaturing PAGE for analysis. The gel was scanned by FujiFilm FLA-9000 Gel Imager in the FAM channel.

### ISCH imaging

The GTFH probe was synthesized as described previously by us (Supplementary Figure S23) (24). HeLa cells were chosen for this assay due to their strong adhesion properties. Alternatively, other cell lines, such as HEK293T cells, which exhibit great transfection efficiency, may also be used. Nevertheless, special care should be taken when handling HEK293T cells as they are less adherent, which could result in cell floating and potential cell loss during processing steps in the assay. HeLa cells were cultured on a 96-well plate with a glass bottom (MatTek). After 24 h, 10 pmol of FAM-c-*kit* 1-T (and Anti-c-*kit* 1) was transfected using Lipofectamine 3000 (Invitrogen, USA) for over 3 h. Then, the medium was removed and L-Apt12-6/PDS (10 μM) was transfected and incubated for 24 h. The cells were then fixed using the same procedure as we reported before (24). After fixation, ISCH-AT was added for hybridization at 37°C overnight. The FV3000 laser scanning confocal microscope (Olympus) with a 60× objective lens was used to obtain fluorescence images, which were then analyzed with Imaris software (Bitplane Corp.).

### Cell imaging

Authenticated and mycoplasma-free HEK293T cells were cultured in DMEM medium (Gibco) supplemented with 10% heat-inactivated fetal bovine serum (Gibco). Each well of a 6-well plate was seeded with 0.5 million cells and grew overnight. Then, 300 nM FAM-labelled L-Apt12-6 was transfected into cells by mixing with Lipofectamine 2000 (Invitrogen, USA). After 12 h of incubation, cells were stained with 5 μg/ml Hoechst 33342 (Sigma Aldrich, USA) at 37°C for 15 min. Fluorescence imaging was performed on a Laser Confocal Scanning Microscope (Leica SPE) with a 60× objective lens. To compare the efficiency of different transfection reagents, HEK293T/Hela/HGC-27 cells were seeded in 96-well plates with 5000 cells/well and grew overnight. Then 100 nM FAM-labelled L-Apt12-6 was transfected into cells by mixing with Lipofectamine 2000/Lipofectamine 3000/PBS control. After 12 h of incubation, cells were stained with 5 μg/ml Hoechst 33342 at 37°C for 15 min. Fluorescence imaging was performed with a 10 × objective lens.

### Cell counting Kit-8 (CCK-8) cytotoxicity assay

HEK293T, HGC-27 and HeLa cells were seeded in a 96-well plate in DMEM with 5000 cells/well and incubated at 37°C for 24 h. Then different concentrations of L-Apt12-6 (0–1000 nM) were transfected into cells using Lipofectamine 2000. After 48 h of incubation, 10 μl of CCK-8 solution (MedChemExpress, USA) was added to each well and incubated at 37°C for 1 h. After that, absorbance at 450 nm was measured using a Molecular Devices SpectraMax ID5 Microplate Reader.

### Dual luciferase reporter gene assay

Wildtype/mutant c-*KIT* constructs (Supplementary Table S1) were inserted into the HSV-TK promoter at the Sac II restriction enzyme site of the Firefly/Renilla dual-luciferase reporter vector, psiCHECK-2 (Promega, USA). Wild-type and mutant reporter plasmids were transfected into HEK293T cells along with L-Apt12-6 (0, 100, 200 and 300 nM) or BRACO-19 (0, 1, 5 and 10 μM) using Lipofectamine 2000. Each well of the 96-well black-wall optical plate was transfected with 10 ng plasmid. After 48 h of incubation, the medium was removed, and the cells were washed with PBS. Then, 20 μl of passive lysis buffer was added and shaken at RT for 20 min. The Dual-Luciferase Reporter Assay kit (Promega, USA) was used to perform luciferase reporter gene assays. The luciferase activity was measured using a Molecular Devices SpectraMax ID5 Microplate Reader.

### Western blot analysis

Each well of a 6-well plate was seeded with 1 million HGC-27 cells in DMEM. After 24 h of incubation, 0–500 nM of L-Apt12-6 or L-SL1 RNA was transfected into the cells using Lipofectamine 2000. PDS was added directly to the medium. Cells were harvested 24 h after transfection and lysed using RIPA lysis buffer (Invitrogen, USA) with proteinase inhibitor (Invitrogen, USA). Protein quantity was measured by UV spectrometry at 595 nm. The protein samples were then transferred to Western blot analysis with c-KIT Rabbit monoclonal antibody (Abcam, UK) and GAPDH Mouse monoclonal antibody (Santa Cruz Biotechnology, USA). Images were taken using a ChemiDoc Touch imaging system (Bio-Rad, USA).

### Total RNA extraction and qRT-PCR test

The same procedures and incubation time as described above for the reporter gene assay and Western blot assay were followed during the transfection process. After incubation, HEK293T or HGC-27 cells were harvested, and total RNAs were extracted using the RNeasy Plus Mini Kit (Qiagen, USA) based on the manufacturer's manual. Reverse transcription was performed using 100 ng of total RNA and PrimeScript RT Master Mix (Takara) in a 20 μl reaction. Next, 1 μl of cDNA solution from the reverse transcription step was used directly for qPCR testing. A 10 μl reaction containing cDNA, SsoAdvanced Universal SYBR Green Supermix (Bio-Rad, USA), and corresponding primers for target mRNA was conducted using a Bio-Rad CFX96 Touch™ Real-Time PCR Detection System (USA).

## Results

### Selection of a novel RNA aptamer for L-c-*kit* 1 dG4 using G4-SELEX-Seq

The parallel G4 conformation is the most abundant and biologically relevant G4 form of DNA (25). Crystal structures for some parallel dG4s have been solved, allowing us to select a stable one as a target (26). Due to the high structural similarity of parallel G4s, we anticipate it is feasible to obtain a parallel G4-specific L-RNA aptamer by using a parallel dG4 as the target in SELEX. We performed *in vitro* SELEX coupled with next generation sequencing (NGS), also referred to as G4-SELEX-Seq (Figure 1C). The target we chose for selection process was a well-studied parallel dG4 named c-*kit* 1 or c-*kit87up*, a 22-nt sequence in the promoter domain of the human proto-oncogene c-*KIT* (Figure 1B) (Supplementary Table S1) (27). It is known that two chiral partners interact through shape complementarity, and thus have reciprocal chiral substrate specificity (28,29). Based on this principle, the D-RNA aptamer was initially selected from a D-RNA library against the enantiomeric form, L-c-*kit* 1 dG4 target, to allow the use of DNA/RNA polymerase during the SELEX process. L-c-*kit* 1 dG4 was labelled with a biotin group at 5′ end, which enabled downstream immobilization onto streptavidin-coated magnetic beads. G4-SELEX-Seq was started from an ssDNA library with a random 40-nucleotide region (N40), working as a template for the dsDNA library. The D-RNA library obtained from *in vitro* transcription was subjected to negative selection with tRNA-blocked beads to remove bead-bound RNA sequences. The bead-unbound RNA library was then incubated with biotin-L-c-*kit* 1 dG4 under defined conditions (Supplementary Table S2). D-RNAs bound to biotin-L-c-*kit* 1 dG4 were eluted from streptavidin-coated magnetic beads and reverse-transcribed to cDNA. After PCR amplification, the resulting dsDNA library was transcribed into RNA and subjected to the next round of selection. The stringency of the selection conditions was gradually increased by decreasing the concentrations of Mg^2+^ in buffer, D-RNA library, target L-c-*kit* 1 dG4 and increasing the washing time (Supplementary Table S2). After four rounds of selection, the enriched DNA library obtained from each round was used for NGS to obtain sequences of aptamer candidates for downstream analysis (Figure 1D).

The NGS results showed that some sequences were enriched after four rounds of selection (Supplementary Table S3). We selected sequences 3–20, referred to as Apt1-Apt18 from the 4th selection round, and tested their binding to L-c-*kit* 1 dG4 using electrophoretic mobility-shift assay (EMSA). Apt12 (Sequence 14 in Supplementary Table S3) was shown to be the tightest binder with a *K*_d_ of 33.65 ± 1.6 nM (Supplementary Figure S1). The predicted secondary structure of Apt12 from mFold was displayed in Figure 1E, which consists of three stems and three loops. The 38-nt fixed primer region used in the selection process is boxed in gray. To improve the efficiency of downstream L-RNA synthesis while maintaining similar binding ability, we optimized the aptamer construct by designing a series of truncations and mutations. First, the fixed primer region was removed and the remaining N40 region (Apt12-1) was predicted to be a stem-loop structure with two 3-nt flanking sequences at both the 5′ and 3′ ends (Figure 1E). Apt12-1 retained the binding ability to L-c-*kit* 1 dG4 with a *K*_d_ of 58.71 ± 9.33 nM (Supplementary Figure S1), indicating that the binding motif is located in the N40 region, while the lower *K*_d_ of Apt12 should be due to the further stabilization of Apt12-1 structure by the fixed primer region. Then the two 3-nt flanking ends were deleted from Apt12-1 without affecting its binding affinity, and the *K*_d_ value of the resulting 34-nt Apt12-2 was calculated to be 56.72 ± 4.42 nM (Supplementary Figure S1). To make the sequence even shorter, we further deleted the U-A base pair from the stem of Apt12-2, which had a weaker effect on binding, as the *K*_d_ of Apt12-3 was tested to be 66.75 ± 8.62 nM (Supplementary Figure S1). In addition, we tried deleting the last C-G (Apt12-4) or last two C-G and G-C base pairs (Apt12-5) in stem, both of which resulted to a much higher *K*_d_ value, 95.39 ± 18.55 and 111.7 ± 14.71 nM, respectively, suggesting that these two base pairs are important for stabilizing aptamer structure and should not be removed (Supplementary Figure S1). Finally, the G-U mismatch in the stem of Apt12-3 was replaced with a G-C base pair to strengthen the stem-loop structure, yielding the 32-nt minimal aptamer Apt12-6 (Figure 1E). To further demonstrate the duplex in stem part is required for binding, we designed two additional aptamer constructs: Apt12-6_G4 motif, which completely removes the stem part, and Apt12-6_SM, which introduces mutations to disrupt the duplex structure (Supplementary Table S1). EMSA gels clearly indicated that both Apt12-6_G4 motif and Apt12-6_SM lost their ability to bind to L-c-*kit* 1 dG4, providing further support for the significance of the duplex in binding (Supplementary Figure S2). All downstream experiments were based on the optimized aptamer sequence, Apt12-6.

### Apt12-6 binds to L-c-*kit* -1 dG4 with strong binding affinity

**Figure 2.**
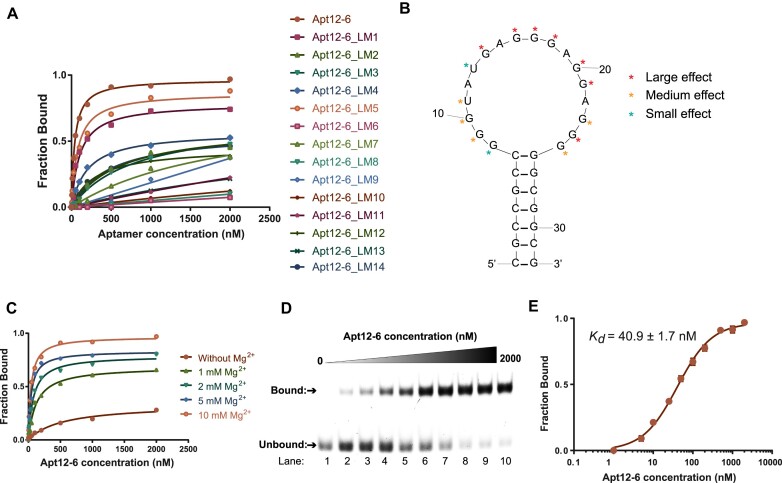
Binding analysis reveals Apt12-6 binds strongly to L-c-*kit* 1 dG4 with nanomolar affinity. (**A**) Mutational analysis of Apt12-6. Fourteen loop mutant (LM) constructs were designed based on the principle of G to A, U to C and C to U single nucleotide substitution in the loop region. The binding results of them showed L-c*-kit* 1 binds strongest to Apt12-6. (**B**) Single nucleotide mutational analysis results of each LM construct based on binding curve from (A). (**C**) Optimization of Mg^2+^ concentration for *in vitro* binding analysis of Apt12-6 to L-c-*kit* 1. *K*_*d*_(5 mM Mg^2+^) = 42.3 ± 5.5 nM. *K*_*d*_(2 mM Mg^2+^) = 78.9 ± 8.1 nM. *K*_*d*_(1 mM Mg^2+^) = 126.1 ± 18.1 nM. A concentration of 10 mM Mg^2+^ was selected for further investigation. (**D**) EMSA shows the binding between Apt12-6 and FAM-L-c-*kit* 1 dG4. With increasing concentration of Apt12-6 (lanes 1–10: 0, 5, 10, 25, 50, 100, 200, 500, 1000, 2000 nM), the unbound band intensity decreases and the bound band intensity increases, indicating the interaction between Apt12-6 and FAM-L-c-*kit* 1 dG4. (**E**) Binding curve of FAM-L-c-*kit* 1 dG4 against Apt12-6 based on data from (D). The *K_d_* is determined to be 40.9 ± 1.7 nM. The error bar represents the standard error of the mean (SEM) of three independent replicates.

To gain more insight into the binding of Apt12-6 to L-c-*kit* 1 dG4, we conducted single nucleotide mutational analysis of the 18-nt loop of Apt12-6. Mutations were introduced in loop regions based on the principle of purine-to-purine (G to A) and pyrimidine-to-pyrimidine substitution (U to C, or C to U) to generate a total of 14 mutant aptamer constructs (Apt12-6_LM1 - Apt12-6_LM14). The EMSA results showed that all constructs exhibited weaker binding ability to L-c-*kit* 1 dG4 than to Apt12-6 (Supplementary Figure S3 and Figure 2A). Specifically, mutations at G14 (Apt12-6_LM6), G16 (Apt12-6_LM7), G17 (Apt12-6_LM8), G18 (Apt12-6_LM9), G20 (Apt12-6_LM10), G21 (Apt12-6_LM11) and G24 (Apt12-6_LM13) caused complete or significant loss in binding affinity to L-c-*kit* 1, and mutations at G9 (Apt12-6_LM2), G10 (Apt12-6_LM3), U11 (Apt12-6_LM4), G23 (Apt12-6_LM12) and G25 (Apt12-6_LM14) displayed medium suppressing effects to binding, while mutations at G8 (Apt12-6_LM1) and U13 (Apt12-6_LM5) showed small effects (Figure 2B). Taken together, these results demonstrate that most nucleotides located in the loop region are critical for binding, so the original loop sequence was retained for Apt12-6 in further analysis.

As an important binding factor, we optimized the Mg^2+^ concentration for *in vitro* binding assays. Binding buffers containing 0, 1, 2, 5 and 10 mM Mg^2+^ were used for the binding reaction, and the results were analyzed using EMSA. Based on the results shown in Supplementary Figure S4 and Figure 2C, the binding was found to be very weak in the absence of Mg^2+^ and became stronger with increasing concentrations of Mg^2+^, indicating that the interactions of Apt12-6 with L-c-*kit* 1 dG4 are Mg^2+^-dependent. A concentration of 10 mM Mg^2+^ provided the strongest binding, which was selected for *in vitro* binding analysis. Under optimal conditions, we tested the binding between Apt12-6 and FAM-L-c-*kit* 1 dG4 by EMSA. As shown in Figure 2D, the unbound band intensity decreased while the bound band intensity increased with the addition of increasing concentrations of Apt12-6 (0–2000 nM), revealing a strong interaction between Apt12-6 and FAM-L-c-*kit* 1 dG4. The binding curve of FAM-L-c-*kit* 1 dG4 against Apt12-6 based on EMSA results is shown in Figure 2E, and the *K*_d_ was determined to be 40.9 ± 1.7 nM.

### Spectroscopic and structure analysis reveals the G4 formation in Apt12-6

**Figure 3.**
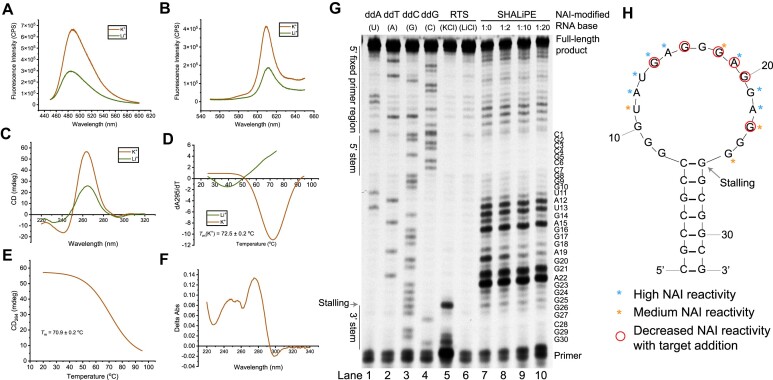
Spectroscopic and structural analysis uncovers Apt12-6 possesses an rG4 structure motif critical for target recognition. (A, B) Fluorescence emission spectra of G4-specific ligand (**A**) ThT (2 μM) and (**B**) NMM (2 μM) with Apt12-6 (500 nM) under 150 mM KCl (red) and LiCl (green) condition. The K^+^-dependent enhanced fluorescence in ThT and NMM supports the formation of rG4 in Apt12-6. (**C**) CD spectrum of 5 μM Apt12-6 under 150 mM KCl (red) and LiCl (green) condition. CD spectra suggests the formation of a parallel G-quadruplex structure in Apt12-6. (**D**) First derivative of the UV melting curve at 295 nm for 5 μM Apt12-6 under 150 mM KCl (red) and LiCl (green) condition. Hypochromic shift was observed, and the melting temperature (*T*_m_) was determined to be 72.5 ± 0.2°C under K^+^ condition. (**E**) CD melting curve of 5 μM Apt12-6 under 150 mM KCl. *T*_m_ was determined to be 70.9 ± 0.2°C. (**F**) Thermal difference spectrum got from the subtraction of the UV absorbance spectrum of Apt12-6 at 20°C from that at 90°C in Supplementary Figure S5C. (**G**) Reverse transcription stalling and SHALiPE probing of Apt12-6ext. Lanes 1–4: Dideoxy sequencing ladder of Apt12-6ext. Lanes 5–6: Reverse transcription stalling observed under KCl condition, but not under LiCl condition, supporting the G4 formation in Apt12-6ext. Lanes 7–10: SHALiPE probing of Apt12-6ext with L-c-*kit* 1 addition (1:0, 1:2, 1:10 and 1:20). (**H**) Predicted secondary structure of Apt12-6 using mFold. The nucleotides with high or medium NAI reactivity of Apt12-6 without target (lane 7) were marked with blue and orange asterisks, respectively. The nucleotides had decreased NAI reactivity with target addition (lanes 8, 9, 10) were marked with red circles.

We found Apt12-6 consists of four tracks of guanine residues, with a score of 0.6731 (>0.5 threshold) using G4NN G4 prediction program (30), suggested that it may form rG4. To verify this, we conducted a series of spectroscopic assays, including fluorescence, circular dichroism (CD) and UV melting assays. All these experiments were conducted under both K^+^ and Li^+^ conditions, considering that rG4 formation is highly dependent on K^+^ but not on Li^+^. Ligand-enhanced fluorescence assays were first performed with two well-studied G4 ligands, Thioflavin T (ThT) (31) and N-methyl mesophorphyrin IX (NMM) (32), which specifically bind to G4 structures and generate enhanced fluorescence. Fluorescence enhancement was observed for both ThT and NMM in the K^+^ condition when compared with the Li^+^ condition (Figure 3A and 3B), suggesting G4 formation in Apt12-6. The CD spectrum of Apt12-6 displayed a positive peak at 265 nm and a negative peak at 240 nm, the characteristic profile for parallel G4 topology (Figure 3C) (33). The CD signal in Li^+^ was much lower than that in K^+^ and the negative peak shifted to 231 nm owing to G4 disruption (Figure 3C). The UV melting curve of Apt12-6 was monitored at 295 nm (Supplementary Figure S5A) and a hyperchromic shift profile was observed under K^+^ (Figure 3D). The melting temperature (*T*_m_) was determined to be 72.5 ± 0.2°C, supporting the formation of a thermostable G4 structure (Figure 3D). To further validate the *T*_m_ value, CD melting at 264 nm (Figure 3E) and UV melting at 260 nm (Supplementary Figure S5B) were conducted. The obtained *T*_m_ values were 70.9 ± 0.2°C and 72.0 ± 0.6°C, respectively, which were similar to the *T*_m_ value got from UV melting at 295 nm. UV absorbance spectra of Apt12-6 were monitored at 20°C and 90°C (Supplementary Figure S5C), generating the Thermal Difference Spectrum (TDS) (Figure 3F). TDS of Apt12-6 exhibited positive peaks at 245 and 274 nm, and a negative peak at 297 nm, the characteristic profile for G4 structure (Figure 3F) (34). Taken together, these distinctive rG4 signals verified the parallel rG4 formation within Apt12-6. To investigate the impact of stem duplex on G4 stability, we tested the UV melting of Apt12-6_G4 motif at 295 nm (Supplementary Figure S5D). Although the *T*_m_ value of Apt12-6_G4 motif (75.6 ± 0.9°C) is slightly higher than that of Apt12-6 (72.5 ± 0.2°C), it showed a broader melting transition from 20 to 90°C, while Apt12-6 remained stable until 55°C, suggesting that the presence of the duplex stem can enhance the stability of the G4 motif at binding temperature (37°C).

To further study the structure of Apt12-6 at single-nucleotide resolution, *in vitro* structure-probing assays were performed. We designed Apt12-6ext, an extended version of Apt12-6 with a primer sequence for reverse transcription purpose (Supplementary Table S1). Dideoxy sequencing and reverse transcriptase stalling (RTS) assays in both KCl and LiCl conditions were first conducted. As shown in Figure 3G, the RNA sequence reads from sequencing lanes 1–4 was consistent with the Apt12-6ext sequence, which served as a sequencing ladder. By inspecting the RTS assay results closely, we observed a strong stalling band in the K^+^ condition (lane 5) at the last G site of the loop of Apt12-6 near the 3′ end, but no stalling was found in the Li^+^ condition (lane 6), suggesting an rG4 structure in the loop of Apt12-6. Next, we also carried out Selective 2′Hydroxyl Acylation analyzed Lithium ion-mediated Primer Extension (SHALiPE) assay to measure the Apt12-6 structure at single nucleotide resolution using 2-methylnicotinic acid imidazolide (NAI). NAI can selectively modify flexible ribonucleotides in single-stranded (ss) RNA regions and block reverse transcriptase elongation, resulting in an NAI modification pattern in denaturing PAGE gel (35). As shown in lane 7 (Figure 3G), the nucleotides in the stem were unreactive with NAI owing to the double-stranded structure, while the nucleotides in the loop, which were supposed to be highly reactive with NAI, showed patterns different from those of ssRNA. Specifically, G8, G9, G10, G16, G17, G20 and G24 had very low or no NAI reactivity, and U11, G18, G23 and G25 showed medium NAI reactivity, revealing that these nucleotides are likely to be involved in a G4 structure, which is consistent with our results above (Figure 3H). When the target L-c-*kit* 1 dG4 was introduced to Apt12-6ext at increasing concentrations, G14, G16, G18, A19, G20 and G23 had reduced NAI reactivity, indicating that L-c-*kit* 1 dG4 likely binds to the G4 of Apt12-6ext and further protects them from the NAI reaction, thus altering the NAI modification patterns (Figure 3G and H). As a control, we added another L-RNA oligo (L-SL1) (Supplementary Table S1) to Apt12-6ext, which cannot interact with Apt12-6 and showed no effect on NAI patterns upon titration (Supplementary Figure S6). These results provide substantial evidence that a G4 structure is formed in the loop region of Apt12-6 and is responsible for its binding to L-c-*kit* 1.

### L-Apt12-6 folds into a left-handed G4 structure and binds to parallel G4 with high affinity and selectivity

**Figure 4.**
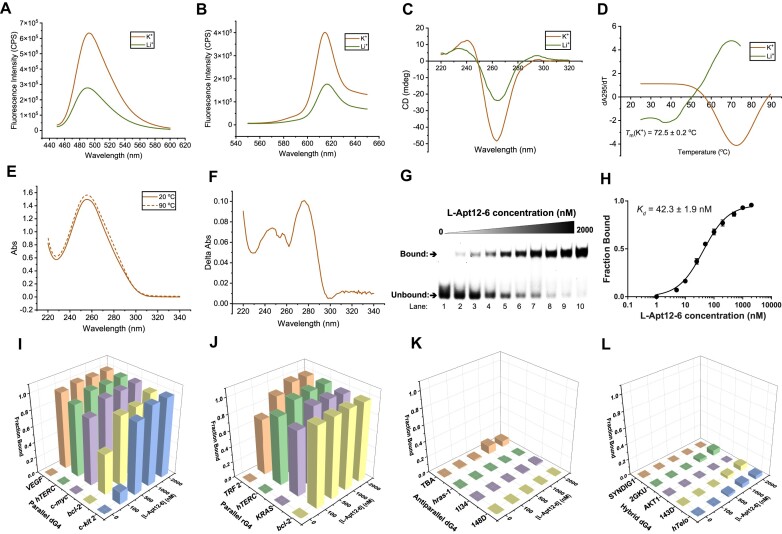
L-Apt12-6 possesses a left-handed G4 structure motif and binds to parallel G4s with high binding affinity and selectivity. (A, B) Fluorescence emission spectra of G4-specific ligand (**A**) ThT (2 μM) and (**B**) NMM (2 μM) with L-Apt12-6 (500 nM) under 150 mM KCl (red) and LiCl (green) condition. The K^+^-dependent enhanced fluorescence in ThT and NMM supports the formation of L-rG4 in L-Apt12-6. (**C**) CD spectrum of 5 μM L-Apt12-6 under 150 mM KCl (red) and LiCl (green) condition, suggesting the left-handed parallel G4 formation in L-Apt12-6. (**D**) First derivative of the UV melting curve at 295 nm for 5 μM L-Apt12-6 under 150 mM KCl (red) and LiCl (green) condition. Hypochromic shift was observed, and the melting temperature (*T*_m_) was determined to be 72.5 ± 0.2°C under K^+^ condition. (**E**) UV absorbance spectra of 5 μM L-Apt12-6 at 20°C and 90°C. (**F**) Thermal difference spectrum got from the subtraction of the UV absorbance spectrum of L-Apt12-6 at 20°C from that at 90°C in (E). (**G**) EMSA shows the binding between L-Apt12-6 and FAM-D-c-*kit* 1 dG4. With increasing concentration of L-Apt12-6 (lanes 1–10: 0, 5, 10, 25, 50, 100, 200, 500, 1000, 2000 nM), the unbound band intensity decreases and the bound band intensity increases, indicating the interaction between L-Apt12-6 and FAM-D-c-*kit* 1 dG4. (**H**) Binding curve of FAM-D-c-*kit* 1 against L-Apt12-6 based on data from (**G**). The *K_d_* is determined to be 42.3 ± 1.9 nM. The error bar represents the SEM of three independent replicates. (I–L) Binding of (**I**) parallel dG4s, (**J**) parallel rG4s, (**K**) antiparallel dG4s and (**L**) hybrid dG4s to increasing concentrations of L-Apt12-6 (0, 100, 500, 1000, 2000 nM). Results suggest that L-Apt12-6 can specifically bind to parallel G4s, but not to antiparallel or hybrid G4s.

After sequence optimization and structure characterization, we converted D-Apt12-6 to L-Apt12-6. L-Apt12-6 is the enantiomeric counterpart of D-Apt12-6, possessing identical physical properties in terms of thermostability and hybridization kinetics (36). Therefore, theoretically, L-Apt12-6 is expected to exhibit the same folding structure as D-Apt12-6. To demonstrate that, we carried out a series of spectroscopic assays, including fluorescence, CD, UV melting and thermal difference spectrum assays. Enhanced fluorescence upon binding to achiral ligands ThT and NMM at K^+^ condition suggested the G4 formation in L-Apt12-6 (Figure 4A and B). CD spectrum of L-Apt12-6 displayed a positive peak at 240 nm and a negative peak at 262 nm, the characteristic profile for left-handed parallel G4 topology (Figure 4C) (22). The UV melting curve of L-Apt12-6 at 295 nm (Figure 4D) exhibited a hyperchromic shift profile in the presence of K^+^. The *T*_m_ was determined to be 72.5 ± 0.2°C, which is consistent with that of D-Apt12-6. UV absorbance of L-Apt12-6 was recorded at 20°C and 90°C (Figure 4E) and generated the thermal difference spectrum of L-Apt12-6 (Figure 4F), which exhibited a similar shape to that of D-Apt12-6, indicating the formation of G4 structure. Then we investigated the binding affinities between L-Apt12-6 and D-c-*kit* 1 dG4. Band shift in EMSA gel exhibited the strong binding of L-Apt12-6 to FAM-D-c-*kit* 1 dG4 (Figure 4G), and the *K*_d_ was determined to be 42.3 ± 1.9 nM (Figure 4H), which is consistent with that of D-Apt12-6 to L-c-*kit* 1 dG4. To corroborate the result, microscale thermophoresis (MST) assay was conducted to double confirm the binding, with a *K*_d_ of 30.7 ± 7.7 nM, further verifying the strong interactions of L-Apt12-6 to D-c-*kit* 1 dG4 (Supplementary Figure S7).

As we initially aimed to select an L-RNA aptamer for parallel G4, we next investigated the G4 conformational selectivity of L-Apt12-6 towards different G4 conformations, including parallel dG4, antiparallel dG4, hybrid dG4 and parallel rG4 (rG4s generally fold into parallel topology). CD spectra of antiparallel and hybrid dG4 sequences were collected to confirm their conformation under K^+^ conditions (Supplementary Figure S8). Binding tests were performed using EMSA (Supplementary Figure S9–S12) and the results showed that L-Apt12-6 can specifically bind to parallel dG4s and rG4s but not antiparallel or hybrid dG4s (Figure 4I-L). The varying binding affinities between L-Apt12-6 and different dG4s and rG4s are due to several factors, including sequence and structural variations, binding site accessibility, as well as thermostability effects. We also investigated the specificity of L-Apt12-6 to non-G4 nucleic acid targets, including two mutant c-*kit* 1 dG4 sequences, poly A/T/C DNAs and DNA/RNA hairpins, none of which showed binding to L-Apt12-6 (Supplementary Figure S13A). Next, to study whether L-Apt12-6 is enantioselective, we tested the binding of the aptamer and the target with the same chirality. No binding was observed between D-Apt12-6 and D-c-*kit*1 or between L-Apt12-6 and L-c-*kit* 1, highlighting that the interaction between the aptamer and target was enantiospecific (Supplementary Figure S13B and C). Taken together, these results demonstrate that G4-SELEX-Seq can be used to develop L-RNA aptamers specific for parallel G4 using a parallel c-*kit* 1 dG4 as the target.

### Control of G4-mediated gene activity *in vitro* and in cells using L-Apt12-6

**Figure 5.**
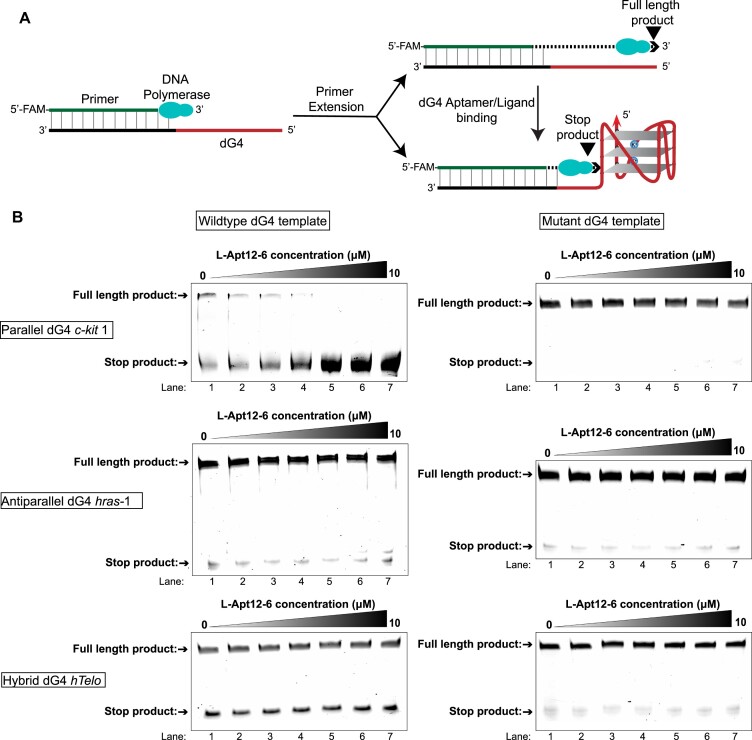
L-Apt12-6 can interact with parallel dG4 to inhibit primer extension. (**A**) Schematic illustration of DNA polymerase stop assay. DNA templates are designed to contain a primer binding region and wildtype/mutant form of parallel, antiparallel or hybrid dG4 sequences. The 5′-FAM labelled primer is extended by Taq DNA polymerase. (**B**) Denaturing PAGE for the DNA polymerase stop assay in the presence of wildtype/mutant parallel dG4 c-*kit* 1, antiparallel dG4 *hras-*1, and hybrid dG4 *hTelo* with increasing concentration of L-Apt12-6 (lanes 1–7: 0, 0.5, 1, 2, 5, 8, 10 μM). L-Apt12-6 can inhibit the full-length product of wildtype c-*kit* 1 template, but not for mutant c-*kit* 1 template, wildtype/mutant *hras*-1 and *hTelo* templates.

To illustrate the applications of L-Apt12-6, we firstly performed DNA polymerase stop assay to study whether the binding of L-Apt12-6 to G4 targets can impair the primer extension process. Primer extension of DNA templates containing G4 can generate full-length and stop products. Aptamer/ligand binding to G4 further inhibits primer extension and generates additional stop products (Figure 5A). As shown in Figure 5B, reaction mixture with the template containing the wild-type parallel dG4 c-*kit* 1 exhibited both full-length and stop products under K^+^, while only full-length products were found under Li^+^ conditions owing to G4 destabilization (Supplementary Figure S14A). With increasing concentrations of L-Apt12-6, wildtype c-*kit* 1 templates got rapid reduction in full-length product, and finally, all the full-length products turned to stop products (Figure 5B). As a control, the primer extension products of templates with mutant c-*kit* 1 that cannot form G4 structures only generated full-length products and no stop products (Figure 5B). The G4-binding ligand BRACO-19 (37) was used as a positive control to incubate with wild-type and mutant c-*kit* 1 templates and showed inhibitory effects on primer extension under K^+^ (Supplementary Figure S14B), indicating that primer extension inhibition is G4-mediated. We also designed templates containing wild-type or mutant antiparallel G4 *hras*-1 and hybrid G4 *hTelo*; no obvious effects were observed with the addition of L-Apt12-6 (Figure 5B). These results reinforced the binding selectivity data in Figure 4, and further suggested that L-Apt12-6 can bind to parallel G4 and inhibit the primer elongation process of the parallel G4 template but has no effect on the non-G4 template or antiparallel/hybrid G4 templates.

Considering its great performance *in vitro*, we next studied whether L-Apt12-6 could target c-*kit* 1 dG4 in cells. We designed a GTFH (G-quadruplex-Triggered Fluorogenic Hybridization) probe for the specific detection of c-*kit* 1 dG4 in cells by employing ISCH (Supplementary Figure S15A) (Supplementary Table S1). ISCH is a G4-specific ligand that generates enhanced fluorescence upon binding to G4s (24). The FAM-c-*kit* 1-T sequence contains the 22-nt c-*kit* 1 dG4 and the 25-nt ‘Tail sequence’ next to c-*kit* 1 with a FAM label at 3′ end (Supplementary Table S1). ISCH-AT is an ‘Anti-Tail Sequence’ with ISCH modified at 3′ end, which can fully hybridize with the Tail sequence of FAM-c-*kit* 1-T and ‘turn on’ the ISCH fluorescence when c-*kit* 1 dG4 structure forms. We transfected FAM-c-*kit* 1-T with L-Apt12-6 or PDS into HeLa cells and stained the cells with ISCH-AT. As shown in Supplementary Figure S15B and C, cells treated with L-Apt12-6 showed 2.02-fold the average ISCH fluorescence intensity of control cells and 1.58-fold the intensity of cells treated with PDS, indicating that L-Apt12-6 can bind c-*kit* 1 dG4 in cells and show a stronger effect than PDS. To further explore the L-RNA aptamer-induced duplex-quadruplex transition in cells, we synthesized Anti-c-*kit* 1, the antisense oligo of c-*kit* 1 dG4, and transfected the duplex into cells (Supplementary Table S1, Supplementary Figure S16A). As shown in Supplementary Figure S16B and C, the ISCH fluorescence in the control group was mostly quenched owing to duplex formation, whereas the fluorescence recovered to 2.77-fold with L-Apt12-6 treatment. PDS treatment led to 1.41-fold ISCH fluorescence recovery, which was not as significant as L-Apt12-6. These results underscored that L-Apt12-6 can target c-*kit* 1 dG4 in cells and control duplex-G4 structural conformation transition.

**Figure 6.**
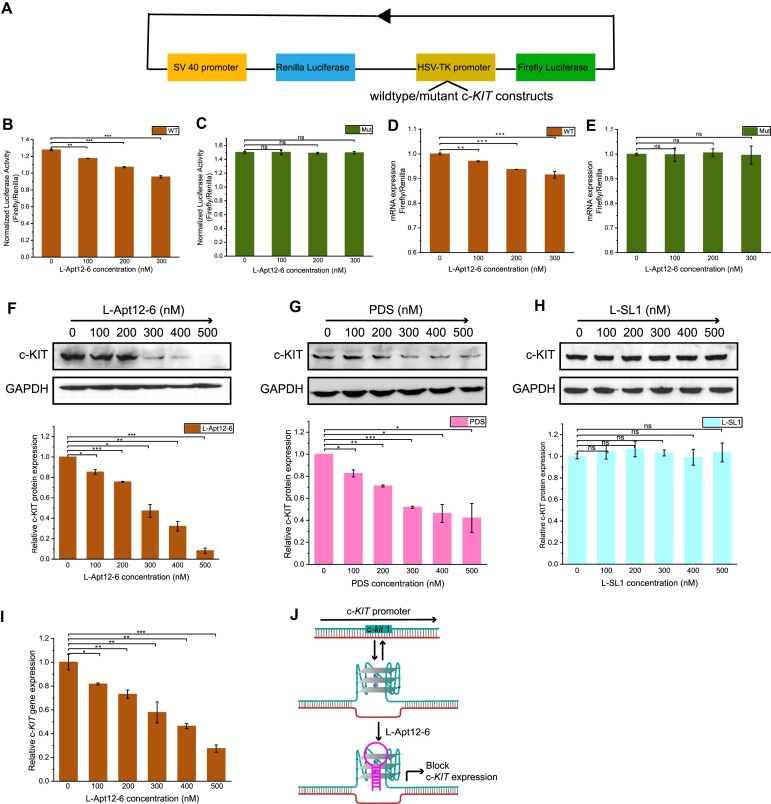
L-Apt12-6 can regulate gene activity in cells. (**A**) Schematic illustration of the design of luciferase reporter plasmids. The wildtype or mutant c-KIT construct is inserted into the HSV-TK promoter of Firefly luciferase. (**B**) Normalized luciferase activity of wildtype c-KIT plasmid (WT) decreases with addition of 0 nM, 100 nM, 200 nM and 300 nM L-Apt12-6. (**C**) Normalized luciferase activity of mutant c-KIT plasmid (Mut) has no obvious change with addition of L-Apt12-6. (**D**) Relative luciferase mRNA expression levels of WT decrease with addition of L-Apt12-6. (**E**) Relative luciferase mRNA expression levels of Mut have no obvious change with addition of L-Apt12-6. (**F**) Western blotting shows endogenous c-KIT expression in HGC-27 cells decreases with L-Apt12-6 treatment. (**G**) Western blotting shows endogenous c-KIT expression in HGC-27 cells decreases with PDS treatment. (**H**) Western blotting shows endogenous c-KIT expression in HGC-27 cells has no obvious change with L-SL1 treatment. The Western blotting results are quantified by ImageJ. (**I**) QPCR result shows relative c-KIT mRNA expression level normalized to house-keeping gene GAPDH decreases with L-Apt12-6 treatment. (**J**) Schematic illustration of L-Apt12-6 binding to c-KIT promoter G4 blocks c-KIT gene transcription and translation. The error bar represents the SEM of three independent replicates. *P< 0.05, **P< 0.01, ***P< 0.001, ns: not significant.

After confirming the binding of L-Apt12-6 to transfected c-*kit* 1 dG4 in cells, we sought to explore whether L-Apt12-6 can be delivered to the cell nucleus as endogenous c-*KIT* DNA is in the nucleus (24). FAM-labelled L-Apt12-6 was transfected into HEK-293T cells and clearly visualized in cell nuclei using Hoechst 33342 staining (Supplementary Figure S17). This indicates that L-Apt12-6 can be transfected into cell nuclei and has the potential for biological applications. Furthermore, L-Apt12-6 can be transfected into HEK293T, HeLa and HGC-27 cells using both Lipofectamine 2000 and 3000 with very high efficiency (Supplementary Figures S18–S20). CCK-8 assays (Supplementary Figure S21) demonstrated that L-Apt12-6 exhibits minimal toxicity towards HEK293T, HGC27 and Hela cells at concentrations of up to 600 nM. Next, we investigated whether L-Apt12-6 can be used to control gene activity in cells using a dual-luciferase reporter gene assay. HEK-293T cells were used here owing to their high transfection efficiency. Wild-type and mutant c-*KIT* constructs were separately inserted into the HSV-TK promoter of the Firefly luciferase. Renilla luciferase was used as an internal control to account for transfection variation (Figure 6A). In the absence of L-Apt12-6, the wild-type construct had lower luciferase activity than the mutant construct (Figure 6B and C), suggesting that the wild-type c-*KIT* sequence can fold into G4 in cells and downregulate gene expression, consistent with other G4-mediated gene suppression reports (22,38). With the addition of L-Apt12-6, the luciferase activity of the wild-type construct decreased significantly, whereas that of the mutant construct did not change (Figure 6B and C), indicating that the suppression of gene expression only occurred for the wild-type c-*KIT* construct. To confirm that the gene downregulation effect was due to the binding of L-Apt12-6 to c-*KIT* promoter G4, a G4-binding ligand, BRACO-19, was used as a positive control to incubate with wild-type and mutant constructs and showed similar results with L-Apt12-6 (Supplementary Figure S22). To study whether L-Apt12-6 regulates gene expression at the transcriptional and/or translational level, we further measured the mRNA levels of Firefly and Renilla luciferase by quantitative reverse transcriptase-polymerase chain reaction (qRT-PCR), and lower expression was observed in wild-type but not mutant constructs (Figure 6D and E), suggesting that L-Apt12-6 can interfere with the transcription process and regulate gene expression at translational level by interacting with c-*kit* 1 dG4.

Based on the regulation activity on reporter gene, we studied the effects of L-Apt12-6 on endogenous c-*KIT* expression in Human Gastric Cancer cell line (HGC-27), which has a high level of c-*KIT* expression (39). HGC-27 cells were treated with 0–500 nM L-Apt12-6 for 24 h, and the levels of c-KIT protein and the housekeeping protein GAPDH were measured by Western blot assays. Figure 6F shows that treatment of HGC-27 cells with L-Apt12-6 led to a dose-dependent reduction in the level of c-KIT protein, which decreased to around 8% when 500 nM L-Apt12-6 was added. As a control, PDS also led to reduction in c-KIT protein expression, although the effects were less significant than those of L-Apt12-6 (Figure 6G). The c-KIT protein level reduced to around 42% when 500 nM PDS was added. In addition, a stem-loop L-RNA oligo (L-SL1) (Supplementary Table S1) that cannot interact with c-*kit* dG4 was used as a negative control and showed no effects on c-KIT expression (Figure 6H). To confirm that the reduction in c-KIT protein levels resulted from the reduction in c-*KIT* mRNA expression, qRT-PCR assays were performed to detect c-*KIT* mRNA levels. The relative c-*KIT* mRNA levels to housekeeping gene GAPDH significantly decreased with the addition of L-Apt12-6 in a dose-dependent manner (Figure 6I). In summary, as a new class of G4 targeting tool, L-Apt12-6 has been demonstrated to bind to the c-*KIT* promoter G-quadruplex in cells and block c-*KIT* gene expression (Figure 6J). This study provides substantial evidence for the suppression of oncogene expression by selective targeting of functional DNA G4 using novel L-RNA aptamer.

## Discussion

L-RNA has attracted increasing attention as a new chemical and biological material owing to its excellent stability and nuclease resistance ability under complex conditions and in cells. Like D-RNA, L-RNA can fold into sophisticated secondary and tertiary structures, with great potential for use as aptamers. L-RNA aptamers have been reported to recognize nucleic acid structural motifs, such as rG4, suggesting that they can be an effective tool for targeting G4 and regulating G4-mediated cell processes (18–21,23). To the best of our knowledge, dG4-targeting L-RNA aptamers have not been reported. Unlike rG4s, which predominately form parallel conformation, dG4s can form parallel, antiparallel and hybrid conformations with high structural similarity, making it especially challenging to target a specific dG4 conformation (2). To develop a robust aptamer-based approach to achieve this goal, we refined the *in vitro* selection strategy by combining it with NGS and developed an innovative selection platform referred to as G4-SELEX-Seq (Figure 1C). Using an important cancer-related parallel dG4, c-*kit* 1, as the target (Figure 1B), we successfully identified an L-RNA aptamer for parallel G4, validating the feasibility and usefulness of G4-SELEX-Seq to screen G4 conformation-selective L-RNA aptamers for the first time.

G4-SELEX-Seq is a process to enrich functional aptamers that can bind to L-dG4 target from a random library, so it is important to analyze the library pool of each selection round and monitor how the sequences population changes during the selection process. Sanger sequencing was used in previous L-RNA aptamer studies to analyze the selection results (18–21,23). Usually, 6–15 selection rounds are required to make the functional sequences highly enriched, and thus, can be recognized from the low sampling of Sanger sequencing (16). However, non-specific binding and PCR problems during the selection process limit the enrichment of functional sequences and decrease the selection efficiency. To solve this problem, we employed NGS as a readout in this study for the first time (Figure 1C). We performed NGS after four selection rounds and obtained all sequence information and the frequency of each round. Two highly enriched sequences (Sequences 1 and 2, Supplementary Table S3) increased much more dramatically than the other sequences during the selection process. They were also found in our other SELEX studies for different targets but without binding to those targets, which are likely enriched by non-specific binding to the components in selection reactions or PCR bias. Therefore, we ignored Sequences 1 and 2, and selected Sequence 3–20 (Supplementary Table S3) for binding verification. Sequence 14 (Apt12) showed the strongest binding among the 18 sequences tested, and was therefore used for further optimization. Interestingly, Apt12 was not observed in our Sanger sequencing results even with up to 12 selection rounds because the small sample size of Sanger sequencing was predominately occupied by non-specific sequences (Supplementary Tables S4 and S5). These findings strongly emphasized that NGS can significantly improve the success rate of SELEX and reduce the number of selection rounds (∼2 days per round) for L-RNA aptamers.

The final 32-nt Apt12-6 sequence was confirmed after a series of construct optimizations (Figure 1E). Apt12-6 adopts a stem-loop structure, as predicted by mFold with an 18-nt loop and 7-bp stem. Single nucleotide mutational analysis of the loop suggested that the guanines in the loop play significant roles in binding and are likely to fold into a G4 structure (Figure 2A and B), which was further confirmed by biophysical (fluorescence, CD, UV/CD melting, TDS), and biochemical assays (RTS, SHALiPE) (Figure 3). The interesting G4-containing structures were also observed in other rG4-targeting L-RNA aptamers (22, 21,20). Our speculation is that the G4 motif is highly suitable for nucleic acid binding due to its ability to form various interactions, such as stacking and hydrogen bonds, with other DNA/RNA sequences. Furthermore, the formation of G4 provided a thermostable structure motif, as illustrated in Figure 3D, for the aptamer as potential molecular recognition element. The stem part stabilizes the loop G4 structure and enables binding, as supported by the results in Supplementary Figure S1 and S2 that shorter stems lead to weaker binding to the target. As a large and complex molecule, L-RNA aptamer is likely to interact with G4s through various mechanisms, such as π-π stacking, electronic interactions, hydrogen bonding, van der Waals forces and shape complementarity. Future investigations using high-resolution structural analysis of L-Apt12-6 and its G4 target can likely provide a clearer understanding of the binding interactions and underlying structural basis.

L-Apt12-6 displayed strong binding affinity and selectivity to parallel G4 over antiparallel/hybrid G4 and other nucleic acid structures (Figure 4 and Supplementary Figure S13), which was further supported by *in vitro* DNA polymerase stop assay (Figure 5). A c-*kit* 1 dG4 specific GTFH probe verified that L-Apt12-6 can target transfected c-*kit* 1 dG4 in cells and control duplex-G4 structural conformation transition, indicating that L-Apt12-6 is correctly folded in the cellular environment (Supplementary Figure S15 and S16). Furthermore, L-Apt12-6 can be delivered into the cell nucleus using Lipofectamine 2000 (Supplementary Figure S17), thus having the potential to target endogenous dG4s in the cell nucleus. Typically, D-DNA/D-RNA can only be transfected into the cell plasma, but with low efficiency in the nucleus owing to nuclease degradation. *In cell* dual luciferase reporter gene assays highlighted the potential utility of L-Apt12-6 to repress transcription and regulate gene activity (Figure 6), while more than 10-fold doses of G4 ligands are required to achieve similar effects (Supplementary Figure S22). Direct targeting of endogenous c-*kit* 1 dG4 inhibited c-*KIT* expression at both the protein and mRNA levels (Figure 6), suggesting that c-*kit* 1 dG4 is a potential drug target. As a control, the inhibitory effects of PDS on endogenous c-*KIT* expression were less significant than those of L-Apt12-6 (Figure 6G), demonstrating that L-Apt12-6 can function as a robust parallel G4 binding tool that is comparable to or even better than G4 ligands in certain application areas. Although the current version of L-Apt12-6 is unable to specifically target single-G4 structures in cells, this limitation can be overcome by incorporating additional modules, such as antisense oligonucleotides, to enhance its specificity. Moreover, L-Apt12-6 can be further modified by attaching other functional proteins, peptides, nucleic acids, or ligands to impart diverse properties and expand its potential applications.

In summary, we developed G4-SELEX-Seq and identified the first L-RNA aptamer to target parallel G4 with high affinity and selectivity. We demonstrated the feasibility and efficiency of our SELEX strategy to identify L-RNA aptamers for targeting G4, and this general platform should be applicable to other nucleic acid structures. Our new and important findings illustrate that L-Apt12-6 can be a valuable tool for selective recognition of G-quadruplex conformation and regulation of G4-mediated gene activity *in vitro* and in cells. With further development and optimization of L-RNA aptamers, such as L-Apt12-6, they can be used in diverse biological and biomedical applications.

## Supplementary Material

gkad900_Supplemental_File

## Data Availability

The data underlying this article are available in the article and in its online supplementary material. Further data will be shared on reasonable request to the corresponding author.
