# Supplementary material for: Selective targeting of parallel G-quadruplex structure using L-RNA aptamer
Source: Nucleic Acids Res. 2023 Oct 23;51(21):11439–52. doi: 10.1093/nar/gkad900 (PMC10681708; doi:10.1093/nar/gkad900)
Supplement: gkad900_Supplemental_File [file gkad900_supplemental_file.docx]

**Supporting Information**

**Selective targeting of parallel G-quadruplex structure using L-RNA aptamer**

Danyang Ji^1^, Jia-Hao Yuan^2^, Shuo-Bin Chen^2^, Jia-Heng Tan^2^ and Chun Kit Kwok^1,3,*^

^1^ Department of Chemistry and State Key Laboratory of Marine Pollution, City University of Hong Kong, Kowloon Tong, Hong Kong SAR, China

^2^ Guangdong Provincial Key Laboratory of New Drug Design and Evaluation, School of Pharmaceutical Sciences, Sun Yat-sen University, Guangzhou, 510006, China

^3^ Shenzhen Research Institute of City University of Hong Kong, Shenzhen, China

*To whom correspondence should be addressed. Tel: +852 3442 6858; Fax: +852 3442 0522; Email: ckkwok42@cityu.edu.hk

**Table of Contents**

**Table S1.** Sequences of the oligonucleotides used in this work.

Table S2. Conditions used for the *in vitro* selection process.

**Table S3.** Next generation sequencing results (top 20 sequences) of 4^th^ selection round.

**Table S4.** Sanger sequencing results of the 20 selected colonies of 7^th^ selection round.

**Table S5.** Sanger sequencing results of the 20 selected colonies of 12^th^ selection round.

**Figure S1.** mFold-predicted structures of Apt12, Apt12-1, Apt12-2, Apt12-3, Apt12-4, and Apt12-5, and their binding affinities to L-c-*kit* 1 tested by EMSA.

**Figure S2.** Binding test of Apt12-6_G4 motif (left) and Apt12-6_SM (right) to FAM-L-c-*kit* 1 using EMSA.

**Figure S3.** Binding test of Apt12-6 loop mutant constructs to FAM-L-c-*kit* 1 using EMSA.

**Figure S4.** EMSA gel of Apt12-6 to FAM-L-c-*kit* 1 under 0, 1, 2, and 5 mM Mg^2+^ conditions.

**Figure S5.** UV spectra for Apt12-6 and Apt12-6_G4 motif.

**Figure S6.** Binding and SHALiPE probing of Apt12-6ext with and without stem-loop L-RNA sequence control.

**Figure S7.** MST binding assay for L-Apt12-6 to FAM-c-*kit* 1 dG4.

**Figure S8.** CD spectra of DNA G4s.

**Figure S9.** Binding test of L-Apt12-6 to parallel dG4s detected by EMSA.

**Figure S10.** Binding test of L-Apt12-6 to parallel rG4s detected by EMSA.

**Figure S11.** Binding test of L-Apt12-6 to antiparallel dG4s detected by EMSA.

**Figure S12.** Binding test of L-Apt12-6 to hybrid dG4s detected by EMSA.

**Figure S13.** Binding selectivity test to non-G4 structures and mirror-image aptamer/target.

**Figure S14.** DNA polymerase stop assay.

**Figure S15.** GTFH probe for c-*kit* 1 dG4 detection.

**Figure S16.** Detection of duplex-quadruplex transitions in HeLa cells.

**Figure S17.** Imaging FAM-L-Apt12-6 in HEK293T cells.

**Figure S18.** FAM-L-Apt12-6 were transfected in HEK293T cells with Lipofectamine 2000 (Lipo2000), Lipofectamine 3000 (Lipo3000) and without transfection reagents.

**Figure S19.** FAM-L-Apt12-6 were transfected in HeLa cells with Lipofectamine 2000 (Lipo2000), Lipofectamine 3000 (Lipo3000) and without transfection reagents.

**Figure S20.** FAM-L-Apt12-6 were transfected in HGC-27 cells with Lipofectamine 2000 (Lipo2000), Lipofectamine 3000 (Lipo3000) and without transfection reagents.

**Figure S21.** Cell Counting Kit-8 (CCK-8) cytotoxicity assays on (A) HEK293T cells, (B) HGC-27 cells and (C) HeLa cells.

**Figure S22.** Reporter gene assay using BRACO-19 treatment.

**Figure S23.** The schematic illustration of GTFH probes synthesis using ISCH-oa1 and azido-modified oligonucleotides.

**Table S1.** Sequences of the oligonucleotides used in this work.

| **Name** | **Sequence (5’-3’)** |
| --- | --- |
| Template for N40  Library | TTCTAATACGACTCACTATAGGTTACCAGCCTTCACTGC(N40) GCACCACGGTCGGTCACAC |
| Forward primer for SELEX | TTCTAATACGACTCACTATAGGTTACCAGCCTTCACTGC |
| Reverse primer for SELEX | GTGTGACCGACCGTGGTGC |
| Biotin-L-c-*kit* 1 | Biotin-AGGGAGGGCGCTGGGAGGAGGG (L-DNA bases) |
| FAM-L-c-*kit* 1 | FAM-AGGGAGGGCGCTGGGAGGAGGG (L-DNA bases) |
| FAM-D-c-*kit* 1 | FAM-AGGGAGGGCGCTGGGAGGAGGG |
| Forward primer for adding NGS linker | TCGTCGGCAGCGTCAGATGTGTATAAGAGACAGXXXXGGTTACCAGCCTTCACTGC (XXXX: barcode) |
| Reverse primer for adding NGS linker | GTCTCGTGGGCTCGGAGATGTGTATAAGAGACAGXXXXGTGTGACCGACCGTGGTGC (XXXX: barcode) |
| Apt12 | GGUUACCAGCCUUCACUGCAGACGUCCGUCGGGUAUGAGGGAGGUGGGGGCGGACGCCAGCACCACGGUCGGUCACAC |
| Apt12-1 | AGACGUCCGUCGGGUAUGAGGGAGGAGGGGGCGGACGCCA |
| Apt12-2 | CGUCCGUCGGGUAUGAGGGAGGAGGGGGCGGACG |
| Apt12-3 | CGCCGUCGGGUAUGAGGGAGGAGGGGGCGGCG |
| Apt12-4 | GCCGUCGGGUAUGAGGGAGGAGGGGGCGGC |
| Apt12-5 | CCGUCGGGUAUGAGGGAGGAGGGGGCGG |
| Apt12-6 | CGCCGCCGGGUAUGAGGGAGGAGGGGGCGGCG |
| Apt12-6_G4 motif | GGGUAUGAGGGAGGAGGG |
| Apt12-6_SM | CACAGACGGGUAUGAGGGAGGAGGGGGCGGCG |
| L-Apt12-6 | CGCCGCCGGGUAUGAGGGAGGAGGGGGCGGCG (L-RNA bases) |
| FAM-L-Apt12-6 | FAM-CGCCGCCGGGUAUGAGGGAGGAGGGGGCGGCG (L-RNA bases) |
| Apt12-6_LM1 | CGCCGCCAGGUAUGAGGGAGGAGGGGGCGGCG |
| Apt12-6_LM2 | CGCCGCCGAGUAUGAGGGAGGAGGGGGCGGCG |
| Apt12-6_LM3 | CGCCGCCGGAUAUGAGGGAGGAGGGGGCGGCG |
| Apt12-6_LM4 | CGCCGCCGGGCAUGAGGGAGGAGGGGGCGGCG |
| Apt12-6_LM5 | CGCCGCCGGGUACGAGGGAGGAGGGGGCGGCG |
| Apt12-6_LM6 | CGCCGCCGGGUAUAAGGGAGGAGGGGGCGGCG |
| Apt12-6_LM7 | CGCCGCCGGGUAUGAAGGAGGAGGGGGCGGCG |
| Apt12-6_LM8 | CGCCGCCGGGUAUGAGAGAGGAGGGGGCGGCG |
| Apt12-6_LM9 | CGCCGCCGGGUAUGAGGAAGGAGGGGGCGGCG |
| Apt12-6_LM10 | CGCCGCCGGGUAUGAGGGAAGAGGGGGCGGCG |
| Apt12-6_LM11 | CGCCGCCGGGUAUGAGGGAGAAGGGGGCGGCG |
| Apt12-6_LM12 | CGCCGCCGGGUAUGAGGGAGGAAGGGGCGGCG |
| Apt12-6_LM13 | CGCCGCCGGGUAUGAGGGAGGAGAGGGCGGCG |
| Apt12-6_LM14 | CGCCGCCGGGUAUGAGGGAGGAGGAGGCGGCG |
| Cy5-Reverse primer for RTS and SHALiPE assay | Cy5-GTGTGACCGACCGTGGTGC |
| Apt12-6ext | GGUUACCAGCCUUCACUGCCGCCGCCGGGUAUGAGGGAGGAGGGGGCGGCGGCACCACGGUCGGUCACAC |
| L-SL1 RNA | GGUUUAUACCUUCCCAGGUAACAAACC (L-RNA bases) |
| FAM*-VEGF* dG4 | FAM-CGGGGCGGGCCGGGGGCGGGGTC |
| FAM-*hTERC* dG4 | FAM-GGGTTGCGGAGGGTGGGCCT |
| FAM-*c-myc* dG4 | FAM-GGAGGGTGGGGAGGGTGGGGAA |
| FAM-*bcl*-2 dG4 | FAM-GGGCGCGGGAGGAAGGGGGCGGG |
| FAM-c-*kit* 2 dG4 | FAM-CGGGCGGGCGCGAGGGAGGGG |
| FAM-TBA dG4 | FAM-GGTTGGTGTGGTTGG |
| FAM-*hras*-1 dG4 | FAM-TCGGGTTGCGGGCGCAGGGCACGGGCG |
| FAM-1I34 dG4 | FAM-GGTTTTGGCAGGGTTTTGGT |
| FAM-148D dG4 | FAM-GGTTGGTGTGGTTGGTT |
| FAM-SYNDIG1 dG4 | FAM-GGATGATGTTGGGCCGGTAGCGGG |
| FAM-2GKU dG4 | FAM-TTGGGTTAGGGTTAGGGTTAGGGA |
| FAM-AKT1 dG4 | FAM-GGGCCGTGGGGCTCCCCGGGCGCTGGG |
| FAM-143D dG4 | FAM-AGGGTTAGGGTTAGGGTTAGGG |
| FAM-*hTelo* dG4 | FAM-TTAGGGTTAGGGTTAGGGTTAGGG |
| FAM-*TRF* 2 rG4 | FAM-CGGGAGGGCGGGGAGGGC |
| FAM-*hTERC* rG4 | FAM-GGGUUGCGGAGGGUGGGCCU |
| FAM-*KRAS* rG4 | FAM-GCGGCGGCGGAGGCA |
| FAM-*bcl*-2 rG4 | FAM-GGGGGCCGUGGGGUGGGAGCUGGGG |
| FAM-PolyA DNA | FAM-AAAAAAAAAAAAAAAAAA |
| FAM-PolyT DNA | FAM-TTTTTTTTTTTTTTTTTT |
| FAM-PolyC DNA | FAM-CCCCCCCCCCCCCCCCCC |
| FAM-Hairpin RNA | FAM-CAGUACAGAUCUGUACUG |
| FAM-Hairpin DNA | FAM-CAGTACAGATCTGTACTG |
| FAM-c-*kit* 1_MT1 | FAM-AAGGAAGGCGCTAGGAGGAAGG |
| FAM-c-*kit* 1_MT2 | FAM-AAAGAAAGCGCTAAGAGGAAAG |
| PSA primer | FAM-ACGACTCACTATAGCAATTGCG |
| PSAtemplate_c-*kit* 1 | AGGGAGGGCGCTGGGAGGAGGGGCCACCGCAATTGCTATAGTGAGTCGT |
| PSAtemplate_c-*kit* 1 _mut | AAAGAAAGCGCTAAGAGGAAAGGCCACCGCAATTGCTATAGTGAGTCGT |
| PSAtemplate_*hras*-1 | TCGGGTTGCGGGCGCAGGGCACGGGCGCGCAATTGCTATAGTGAGTCGT |
| PSAtemplate_*hras*-1_mut | TCAAGTTGCAAGCGCAAAGCACAAGCGCGCAATTGCTATAGTGAGTCGT |
| PSAtemplate_*hTelo* | TTAGGGTTAGGGTTAGGGTTAGGGCGCAATTGCTATAGTGAGTCGT |
| PSAtemplate_*hTelo*_mut | TTAAAGTTAAAGTTAAAGTTAAAGCGCAATTGCTATAGTGAGTCGT |
| FAM-c-*kit* 1-T | AGGGAGGGCGCTGGGAGGAGGGGCTGCTGCTCGCCGCTCGCGGCTCT-FAM |
| Anti-c-*kit* 1 | CCCTCCTCCCAGCGCCCTCCCT |
| ISCH-AT | AGAGCCGCGAGCGGCGAGCAGCAGC-ISCH |
| Wildtype c-*KIT* construct inserted in vector | CCGGGCGGGCGCGAGGGAGGGGAGGCGAGGAGGGGCGTGGCCGGCGCGCAGAGGGAGGGCGCTGGGAGGAGGGGC |
| Mutant c-*KIT* construct inserted in vector | CCAAGCAAGCGCGAAAGAAAGGAGGCGAGGAGGGGCGTGGCCGGCGCGCAGAAAGAAAGCGCTAAGAGGAAAGGC |
| Renilla luciferase forward primer | ACAAGTACCTCACCGCTTGG |
| Renilla luciferase reverse primer | GACACTCTCAGCATGGACGA |
| Firefly luciferase forward primer | GGACATCACCTATGCCGAGT |
| Firefly luciferase reverse primer | GTTCTCAGAGCACACCACGA |
| c-*KIT* forward primer | CGTGGAAAAGAGAAAACAGTCA |
| c-*KIT* reverse primer | CACCGTGATGCCAGCTATTA |
| GAPDH forward primer | GGAGCGAGATCCCTCCAAAAT |
| GAPDH reverse primer | GGCTGTTGTCATACTTCTCATGG |

Table S2. Conditions used for the *in vitro* selection process.

| Selection round | 1 | 2 | 3 | 4 |
| --- | --- | --- | --- | --- |
| MgCl_2_ concentration (mM) | 5 | 5 | 5 | 1 |
| D-RNA library (µM) | 3 | 1 | 0.3 | 0.1 |
| Biotin-L-c-*kit* 1 (µM) | 0.65 | 0.65 | 0.33 | 0.1 |
| Negative selection time (h) | 2 | 2 | 2 | 1 |
| Positive selection time (min) | 30 | 30 | 30 | 30 |
| Washing time (min) | 1 | 1 | 1 | 10 |
| Incubation temperature (^o^C) | 25 | 25 | 37 | 37 |
| PCR cycle | 8 | 8 | 8 | 8 |

**Table S3.** Next generation sequencing results (top 20 sequences) of 4^th^ selection round.

| Sequence number | N40 sequence (5’-3’) | Reads |
| --- | --- | --- |
| 1 | AAGCACGCGGAGGGTAGGGCAAGGGCGGGAGCGTTGCGAC | 2712 |
| 2 | TCCCGTAGGGATTAGGGTGGAGGTGATGTGCGGGGGCAGT | 1749 |
| 3 | GCGGAGTGTTACAGCGGGTGGATGGGGCGGACGCTCGGCA | 824 |
| 4 | GCGCCAGTGCGGGGGGCAGAGCGGAGGGAGAGCGCGCGTG | 262 |
| 5 | GCGGACGCCGGCGGAGACAGGGCGGAGGGAGGGGGCGCGC | 228 |
| 6 | CGAGCCGCATGTACGCGGGGGGGAGGGTGGATGTGAGCTC | 266 |
| 7 | GCTTCCCGGGGGGACGGAGTAGGGAGGAGGCCCGGACGGC | 206 |
| 8 | GGGTGGGTGGGCGGAAGAGTTGTGCTGGTGTGGCGCTTAG | 192 |
| 9 | GAGTACACGTGTGCGTGGGCGGGTCGGGTGGACGTGTAGC | 174 |
| 10 | GGGCGGCGAGACAGTGGGAGGATGGGGAGGCGTCGTCCGG | 147 |
| 11 | TATGCGCGGCATGTGCGGGAGAGGGGGGAGGACGCGCGTT | 135 |
| 12 | TAATGGCGGGCGGAAGCGGGTGGGTGTTAGACATTGGCGT | 130 |
| 13 | TGTAGCGGATTTCCAGTGGGTGGATGGGGCGGGTCCGCGC | 178 |
| 14 | AGACGTCCGTCGGGTATGAGGGAGGAGGGGGCGGACGCCA | 125 |
| 15 | GCCCGGATAAAGGGGAGAGGTGGATATTAGGGATCCGGGA | 109 |
| 16 | TCTCCTAAGATGTGCGGGCGGAGTGGGTGGCATAGGACGA | 106 |
| 17 | TCAGGTCGCGGGGACTGGGAGGAGGAGATGGGCGGCCAGA | 101 |
| 18 | AGGTCGGGAATATCGTGGGAGAGGGGGGCGGTCCCGACTT | 95 |
| 19 | CACTCGTGGTGCCTCAGGGTTGGGAGTGAGGTGATGGGGG | 95 |
| 20 | CAGGTGCACGCTCGGGATGGGGAGGGGGTGATGAGAACGT | 89 |

Sequence 14 is referred to as Apt12-1, which is highlighted in red.

**Table S4.** Sanger sequencing results of the 20 selected colonies of 7^th^ selection round.

| Sequence number | N40 sequence (5’-3’) | Reads |
| --- | --- | --- |
| 1 | AAGCACGCGGAGGGTAGGGCAAGGGCGGGAGCGTTGCGAC | 15 |
| 2 | TCCCGTAGGGATTAGGGTGGAGGTGATGTGCGGGGGCAGT | 4 |
| 3 | GCTCCGTTATAGGGATGCGAAGGGTGGGAGGTTGCGCGGT | 1 |

**Table S5.** Sanger sequencing results of the 20 selected colonies of 12^th^ selection round.

| Sequence number | N40 sequence (5’-3’) | Reads |
| --- | --- | --- |
| 1 | AAGCACGCGGAGGGTAGGGCAAGGGCGGGAGCGTTGCGAC | 13 |
| 2 | TCCCGTAGGGATTAGGGTGGAGGTGATGTGCGGGGGCAGT | 3 |
| 3 | GCUCCGUUAUAGGGAUGCGAAGGGUGGGAGGUUGCGCGGU | 1 |
| 4 | AGUUCGGAAUAAAGGGUUAGGGUUAGGGAAGGGAUCCGCU | 1 |
| 5 | AGGGAGGGCAGGGCAAGGGAGGCGGAGGCUGCGAGCGAGU | 1 |
| 6 | AAAUGAGGGGGGGAGGGUGGAUGGUCUGUAGUGAUUUUGGU | 1 |


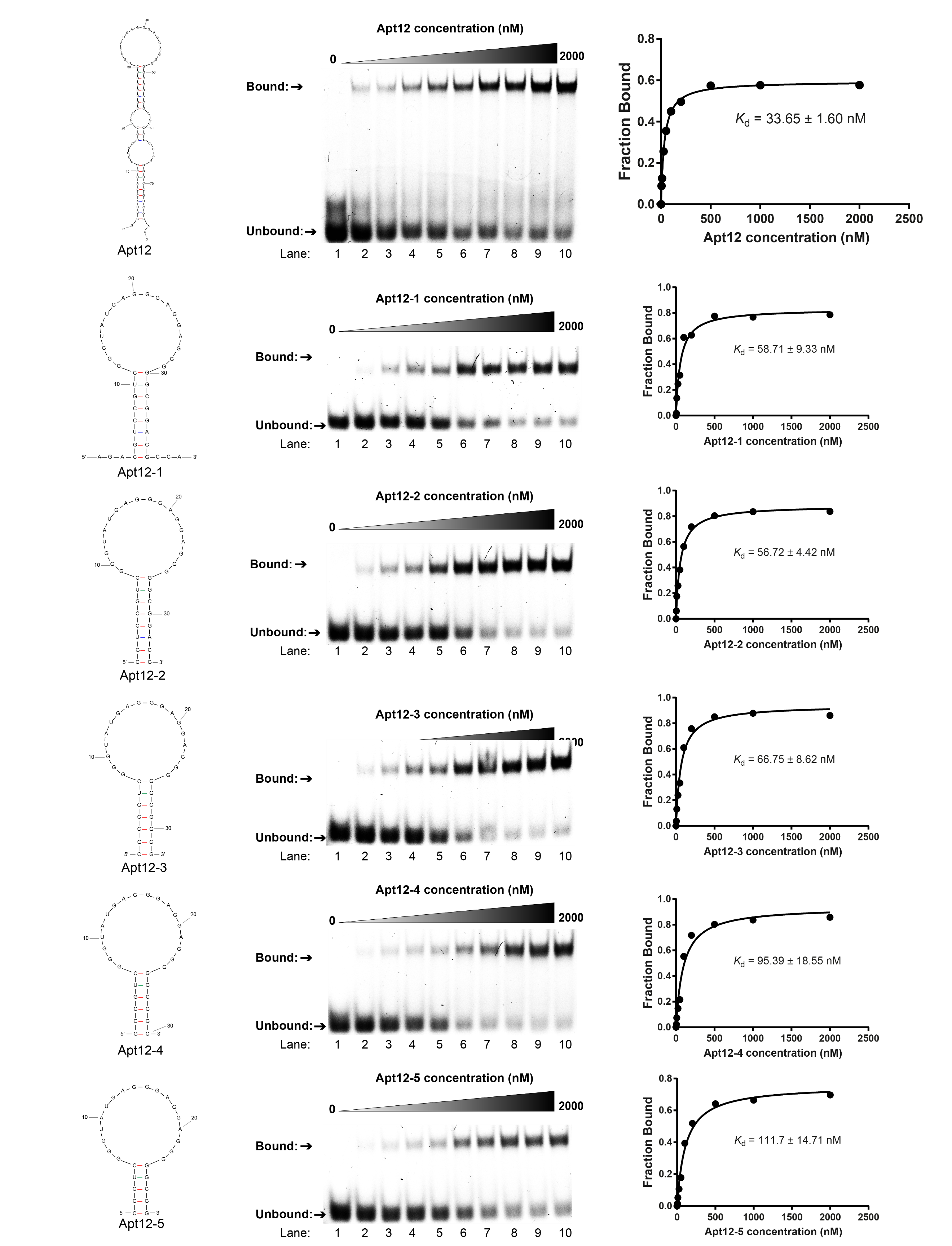


**Figure S1.** mFold-predicted structures of Apt12, Apt12-1, Apt12-2, Apt12-3, Apt12-4, and Apt12-5, and their binding affinities to L-c-*kit* 1 tested by EMSA.


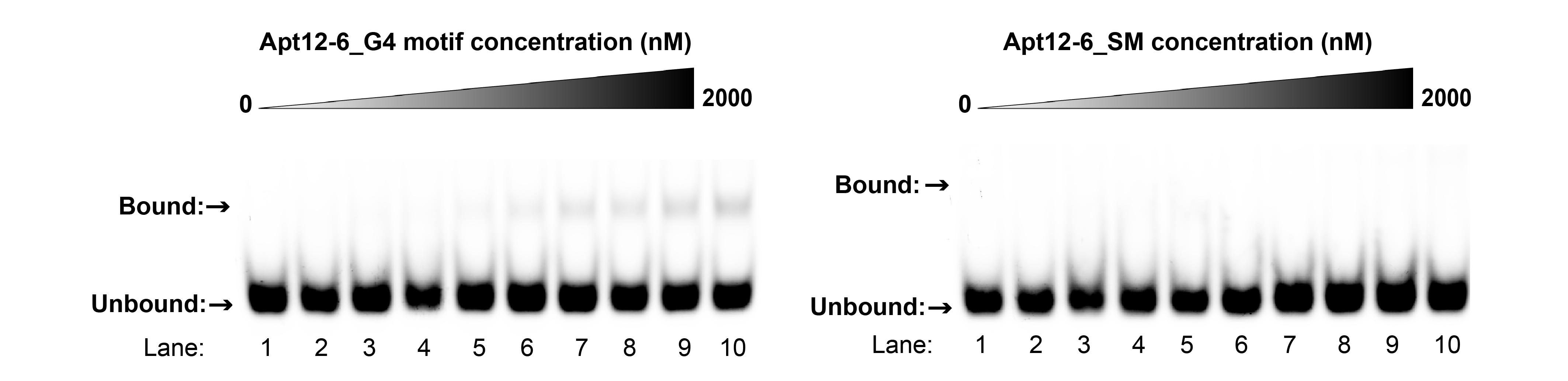


**Figure S2.** Binding test of Apt12-6_G4 motif (left) and Apt12-6_SM (right) to FAM-L-c-*kit* 1 using EMSA. Neither of them shows binding to FAM-L-c-*kit* 1.


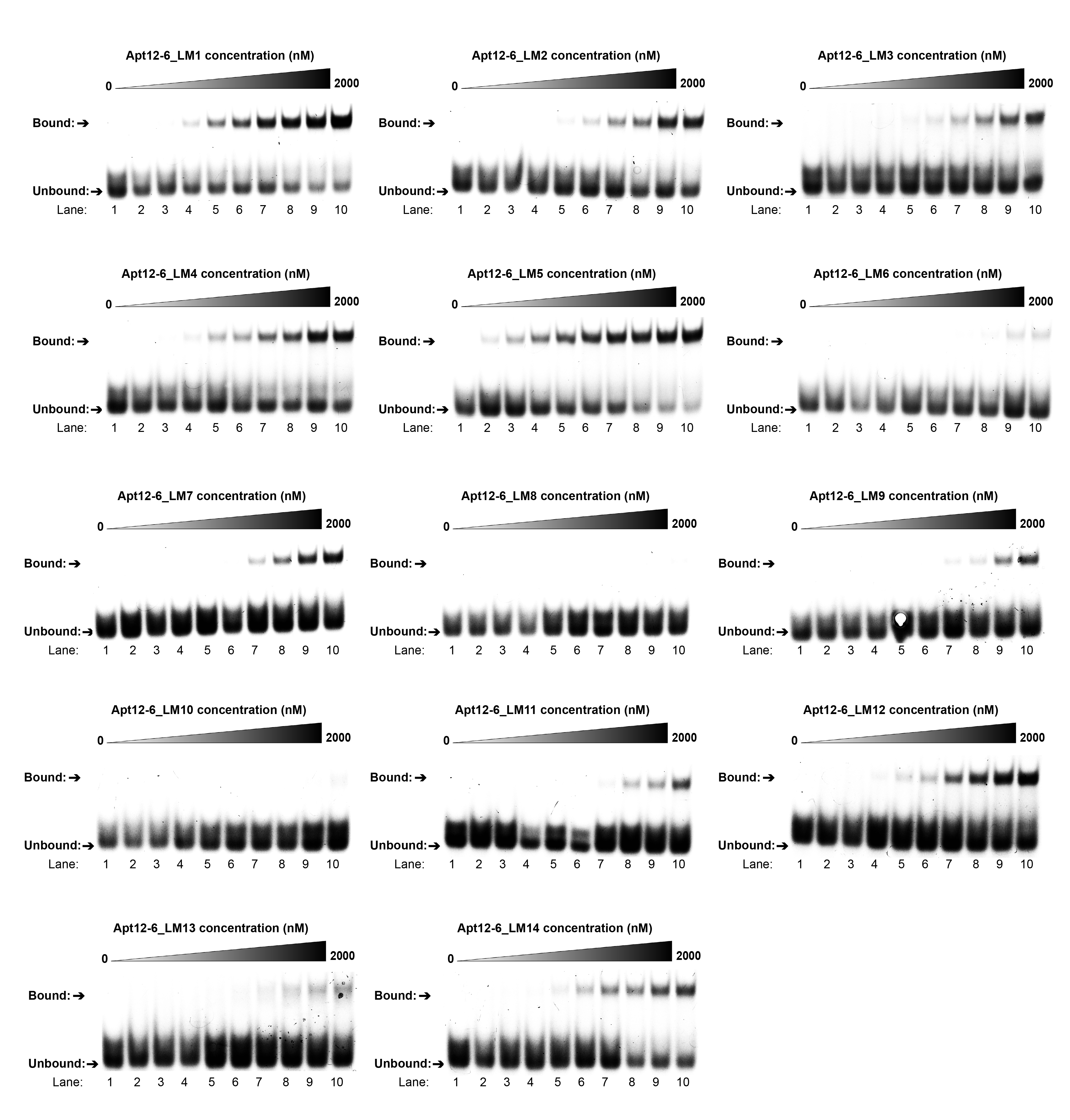


**Figure S3.** Binding test of Apt12-6 loop mutant constructs to FAM-L-c-*kit* 1 using EMSA. Fourteen loop mutant (LM) constructs were designed based on the principle of G to A, U to C, and C to U single nucleotide substitution in the loop region.


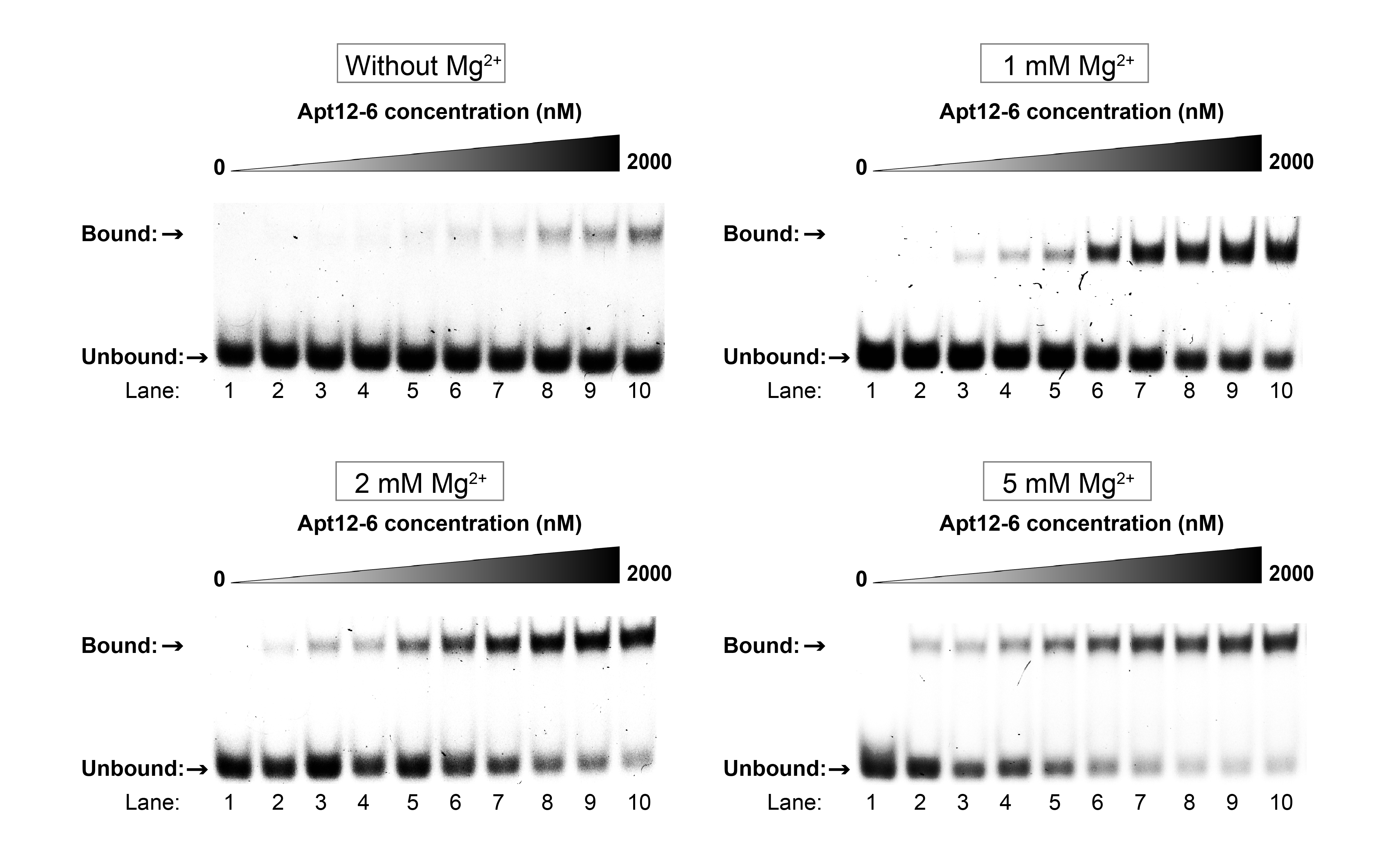
**Figure S4.** EMSA gel of Apt12-6 to FAM-L-c-*kit* 1 under 0, 1, 2, and 5 mM Mg^2+^ conditions.


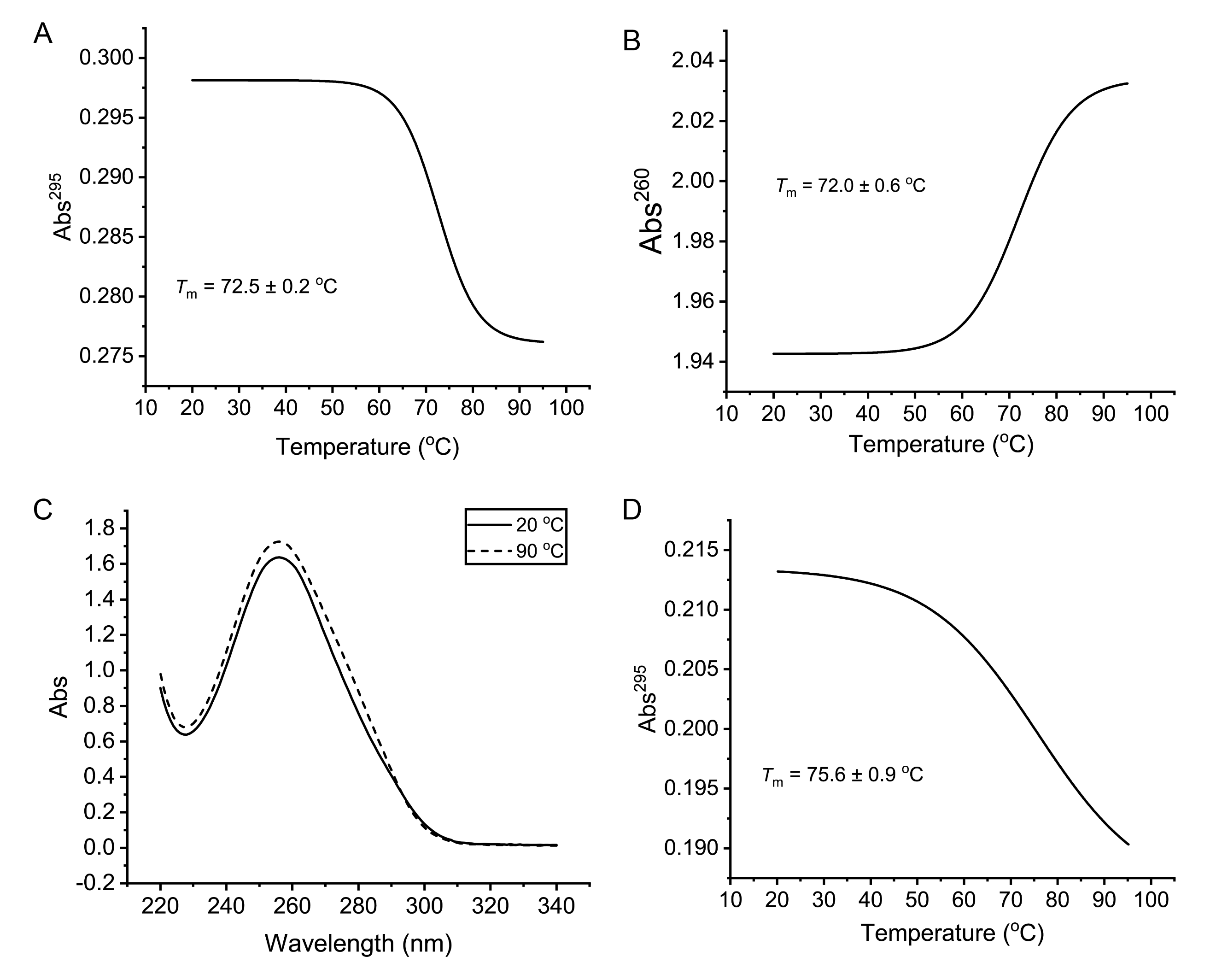


**Figure S5.** UV spectra for Apt12-6 and Apt12-6_G4 motif. (A) UV melting curve of Apt12-6 at 295 nm. *T*_m_ was tested to be 72.5 ± 0.2 ^o^C. (B) UV melting curve of Apt12-6 at 260 nm. *T*_m_ was tested to be 72.0 ± 0.6 ^o^C. (C) UV absorbance spectra of Apt12-6 at 20 ^o^C and 90 ^o^C. (D) UV melting curve of Apt12-6_G4 motif at 295 nm. *T*_m_ was tested to be 75.6 ± 0.9 ^o^C.


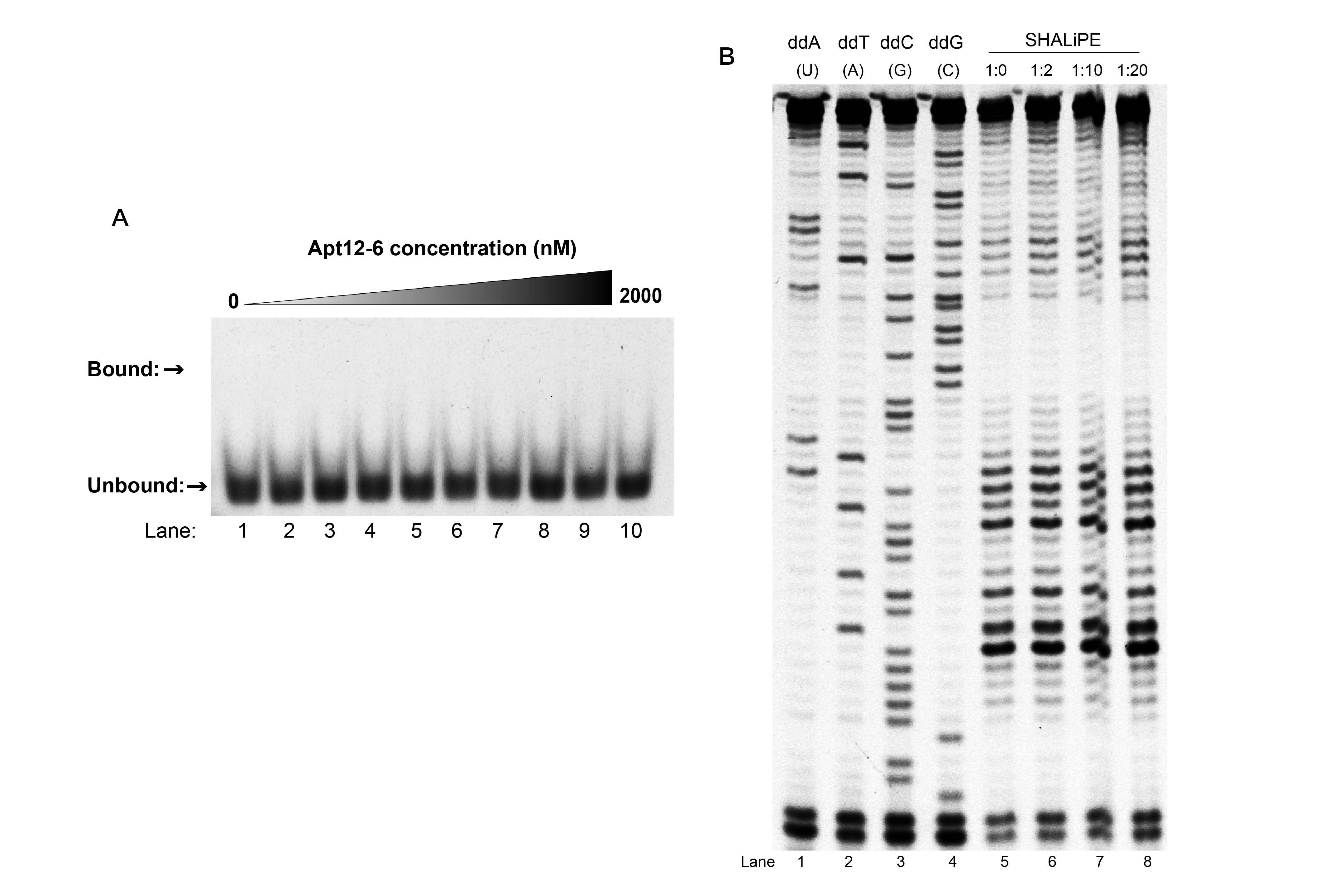


Figure S6. Binding and SHALiPE probing of Apt12-6ext with and without stem-loop L-RNA sequence control. (A) EMSA gel showing that Apt12-6ext does not bind to a stem-loop L-RNA sequence (L-SL1). (B) SHALiPE gel shows that L-SL1 did not affect the NAI patterns of Apt12-6ext. Lanes 1-4: Dideoxy sequencing ladder of Apt12-6ext. Lanes 5-8: SHALiPE probing of Apt12-6ext with L-SL1 as a negative control (1:0, 1:2, 1:10, and 1:20).


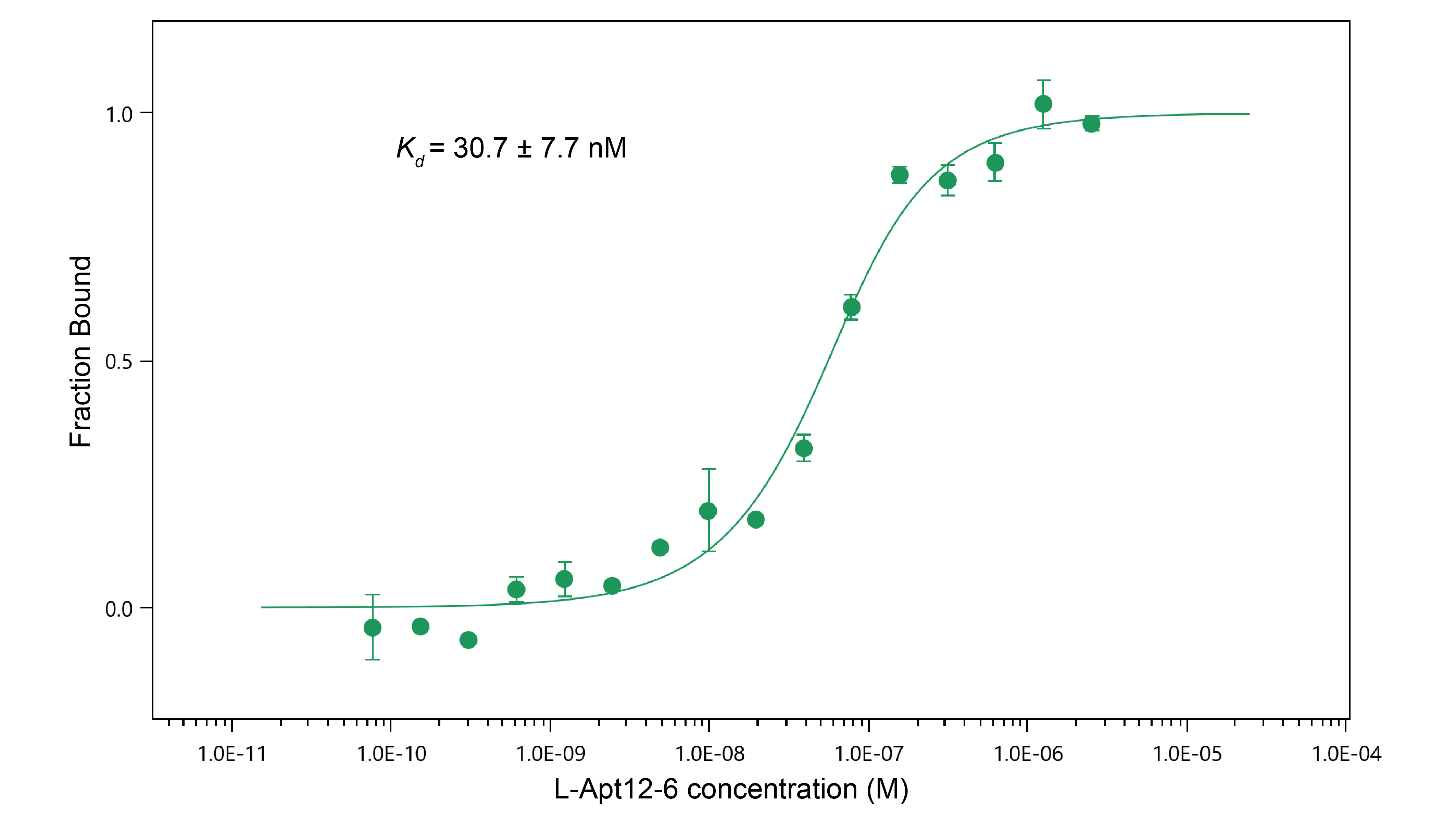


Figure S7. MST binding assay for L-Apt12-6 to FAM-c-*kit* 1 dG4. The *K*_d_ was determined to be 30.7 ± 7.7 nM.

**
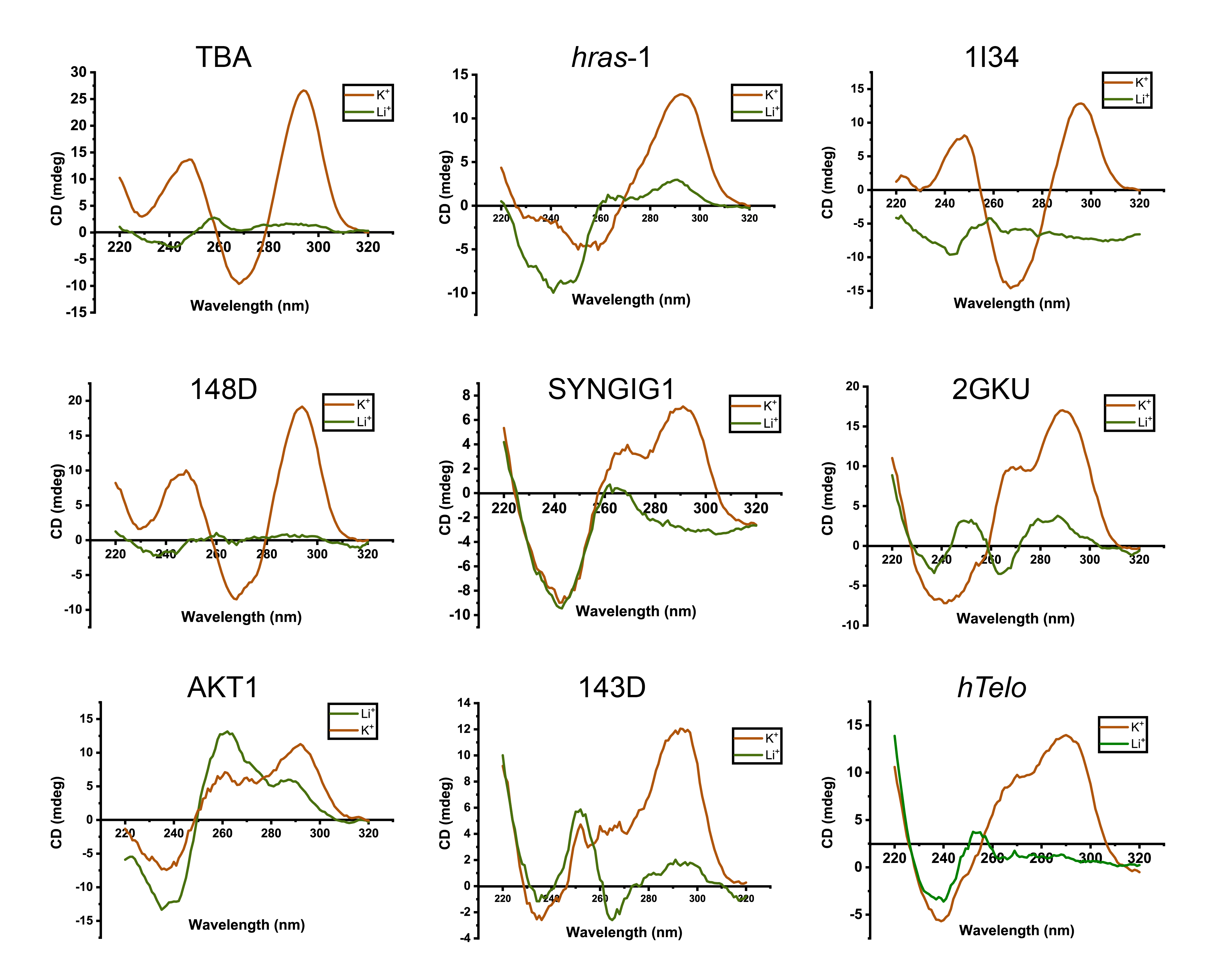
**

**Figure S8.** CD spectra of DNA G4s. The positive peak at 290 nm indicates that TBA, *hras*-1, 1I34 and 148D form antiparallel G4. The positive peaks at 265 nm and 290 nm, and negative peak at 240 nm indicate that SYNGIG1, 2GKU, AKT1, 143D and *hTelo* form hybrid G4.

**
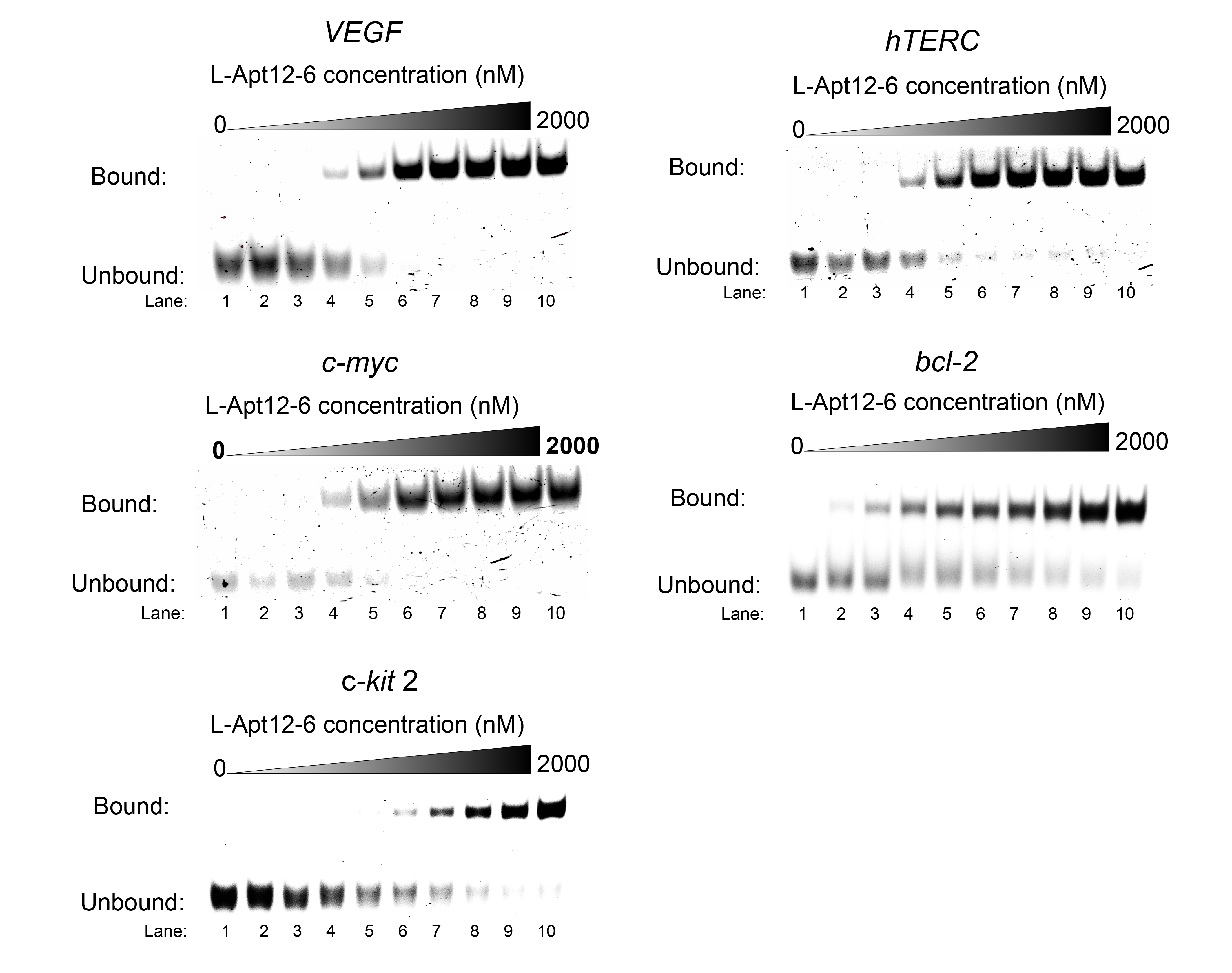
**

**Figure S9.** Binding test of L-Apt12-6 to parallel dG4s detected by EMSA. L-Apt12-6 can bind to all the tested parallel dG4s.

**
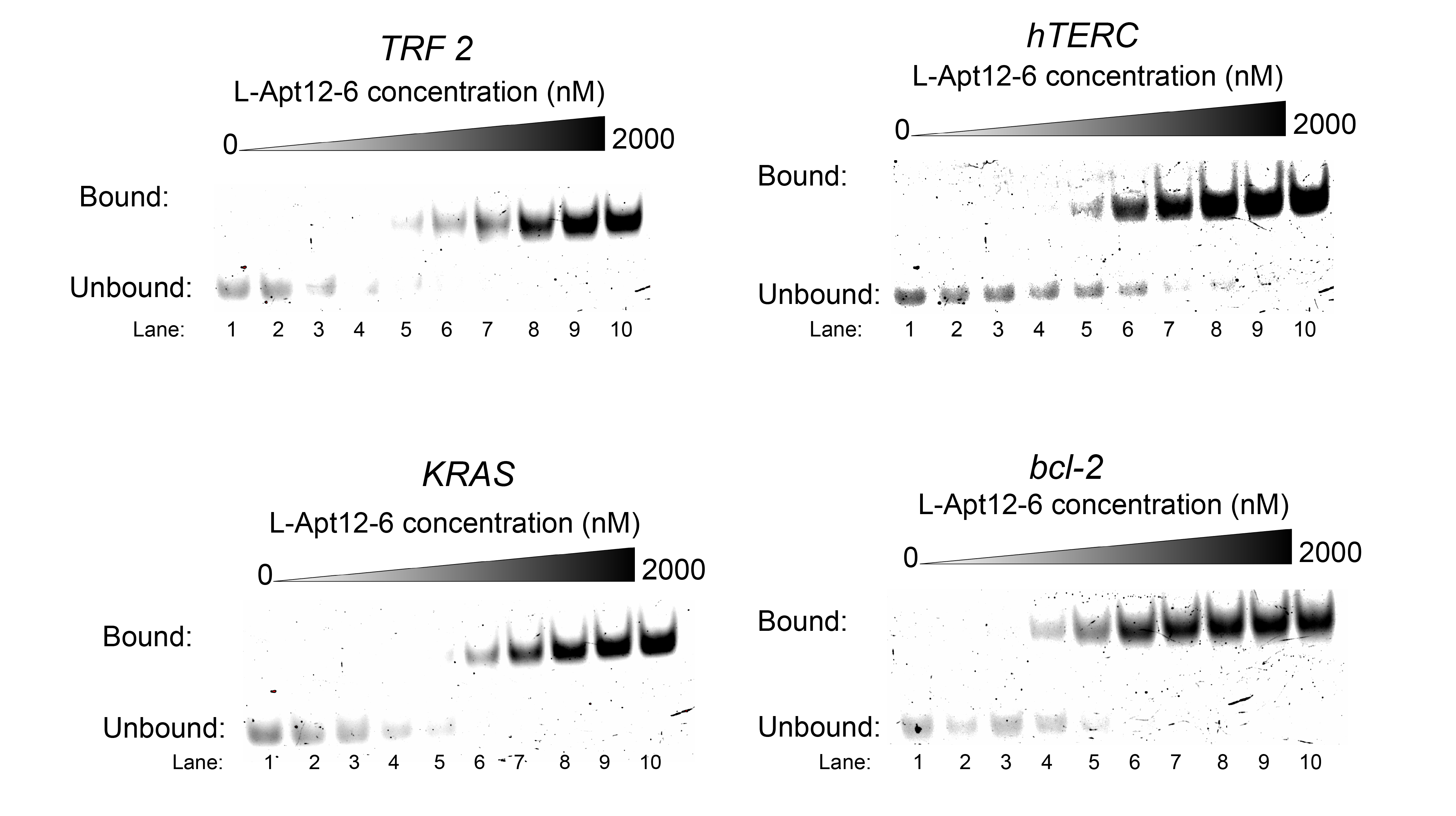
**

**Figure S10.** Binding test of L-Apt12-6 to parallel rG4s detected by EMSA. L-Apt12-6 can bind to all the tested rG4s.

**
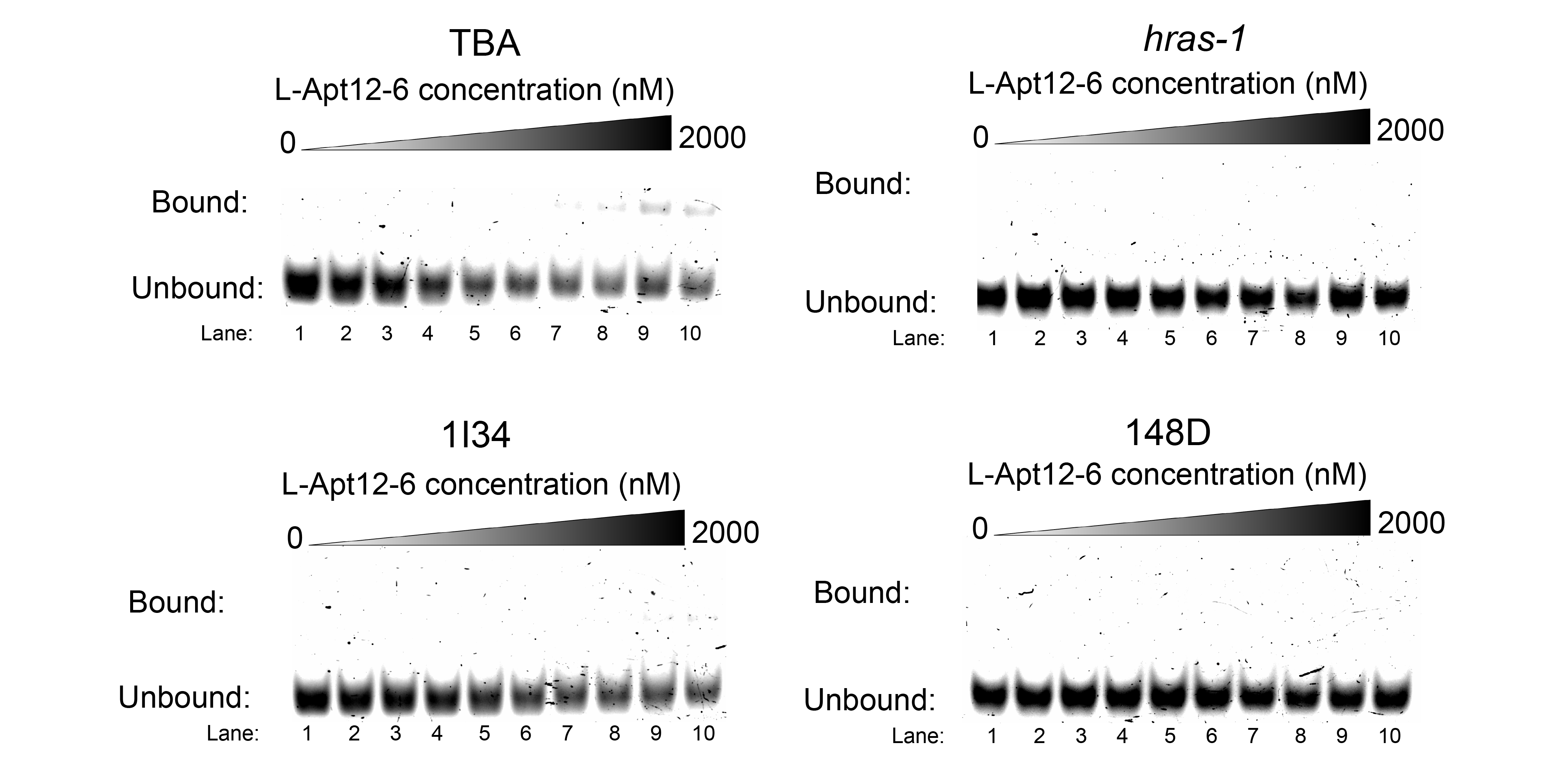
**

**Figure S11.** Binding test of L-Apt12-6 to antiparallel dG4s detected by EMSA. L-Apt12-6 has no binding to all the tested antiparallel dG4s.

**
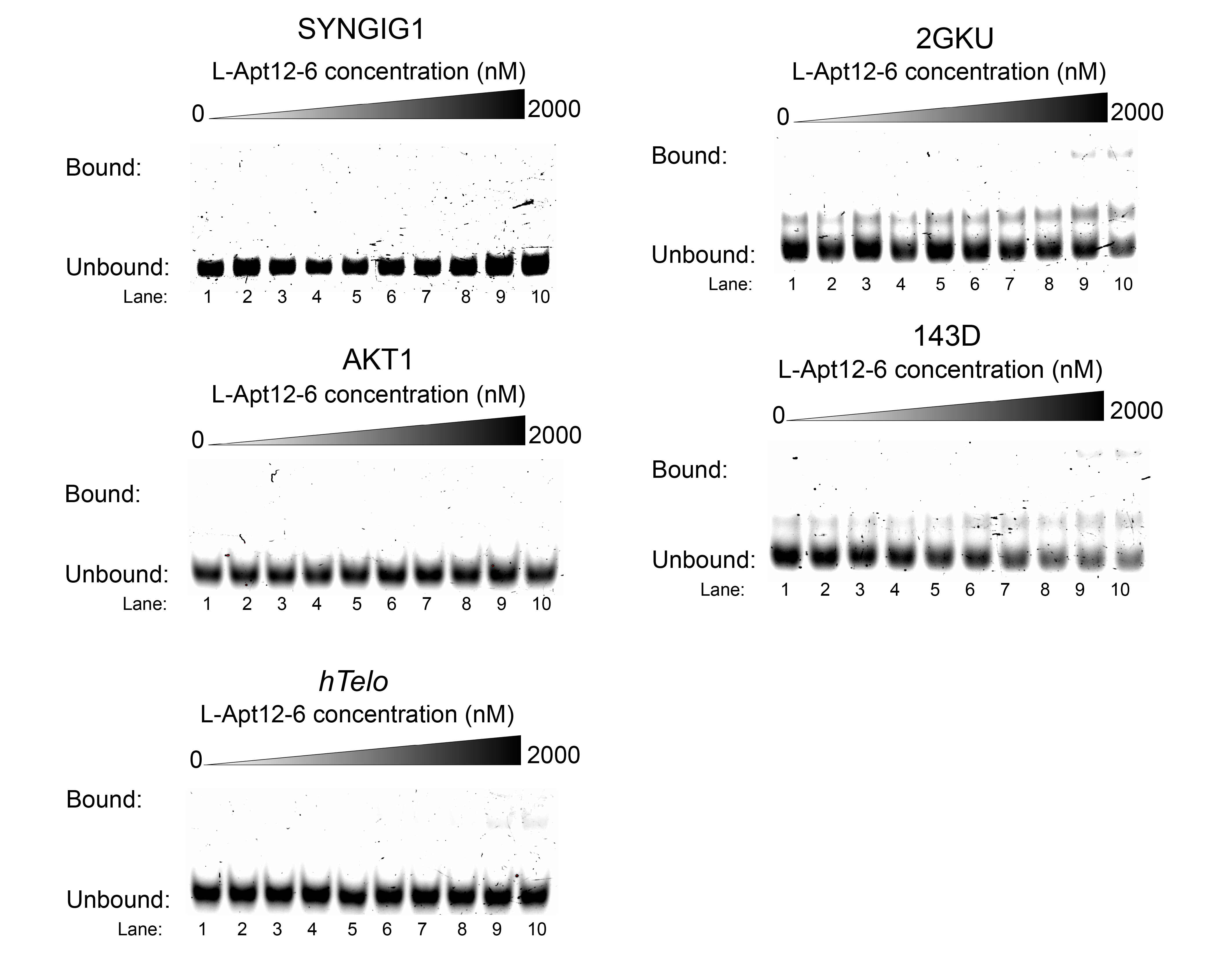
**

**Figure S12.** Binding test of L-Apt12-6 to hybrid dG4s detected by EMSA. L-Apt12-6 has no binding to all the tested hybrid dG4s.

**
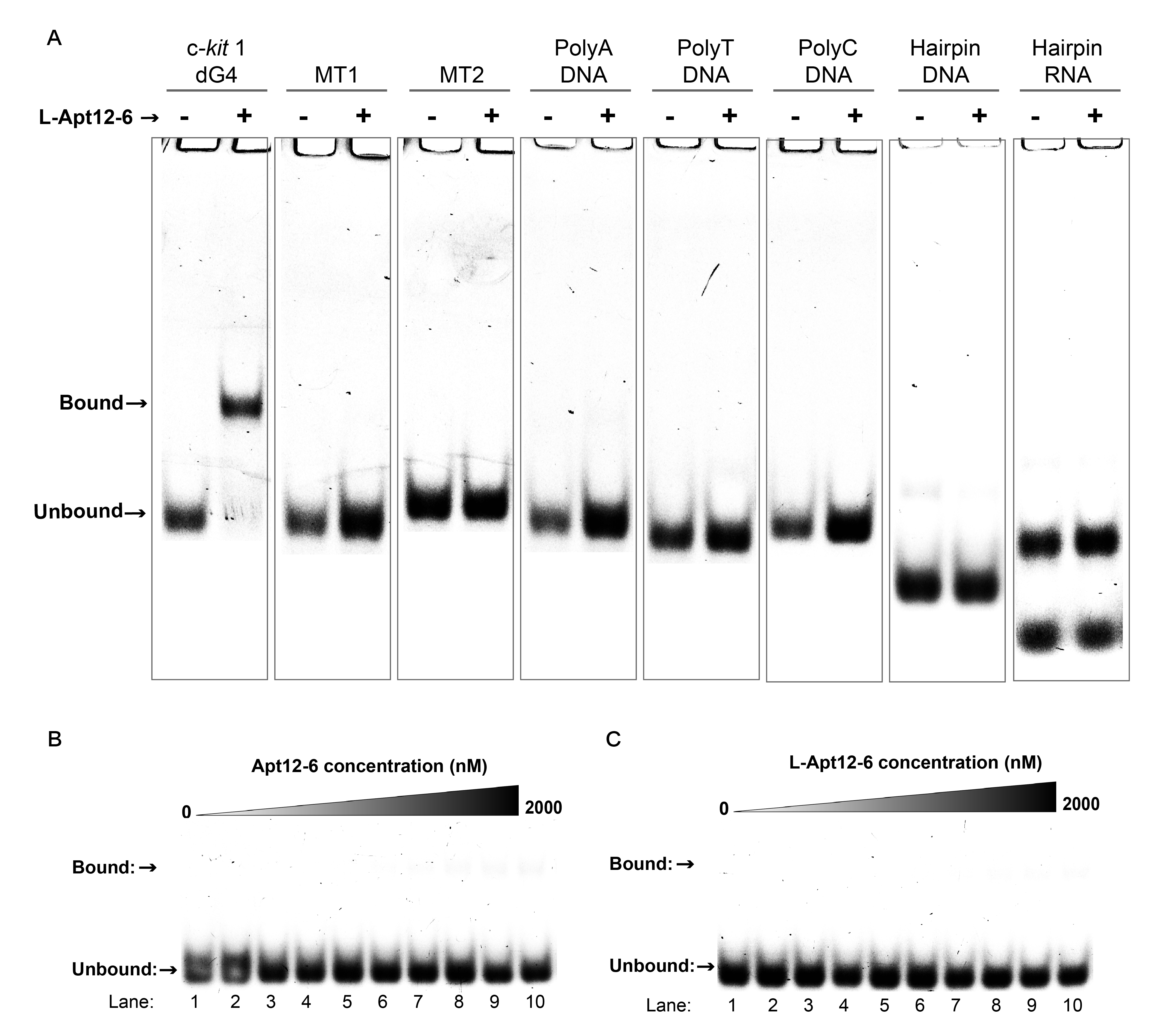
**

**Figure S13.** Binding selectivity test to non-G4 structures and mirror-image aptamer/target. (A) EMSA gel showing that L-Apt12-6 does not bind to non-G4s. (B) EMSA gel showing that D-Apt12-6 cannot bind to D-c-*kit* 1 dG4. (C) EMSA gel showing that L-Apt12-6 cannot bind to L-c-*kit* 1 dG4.

**
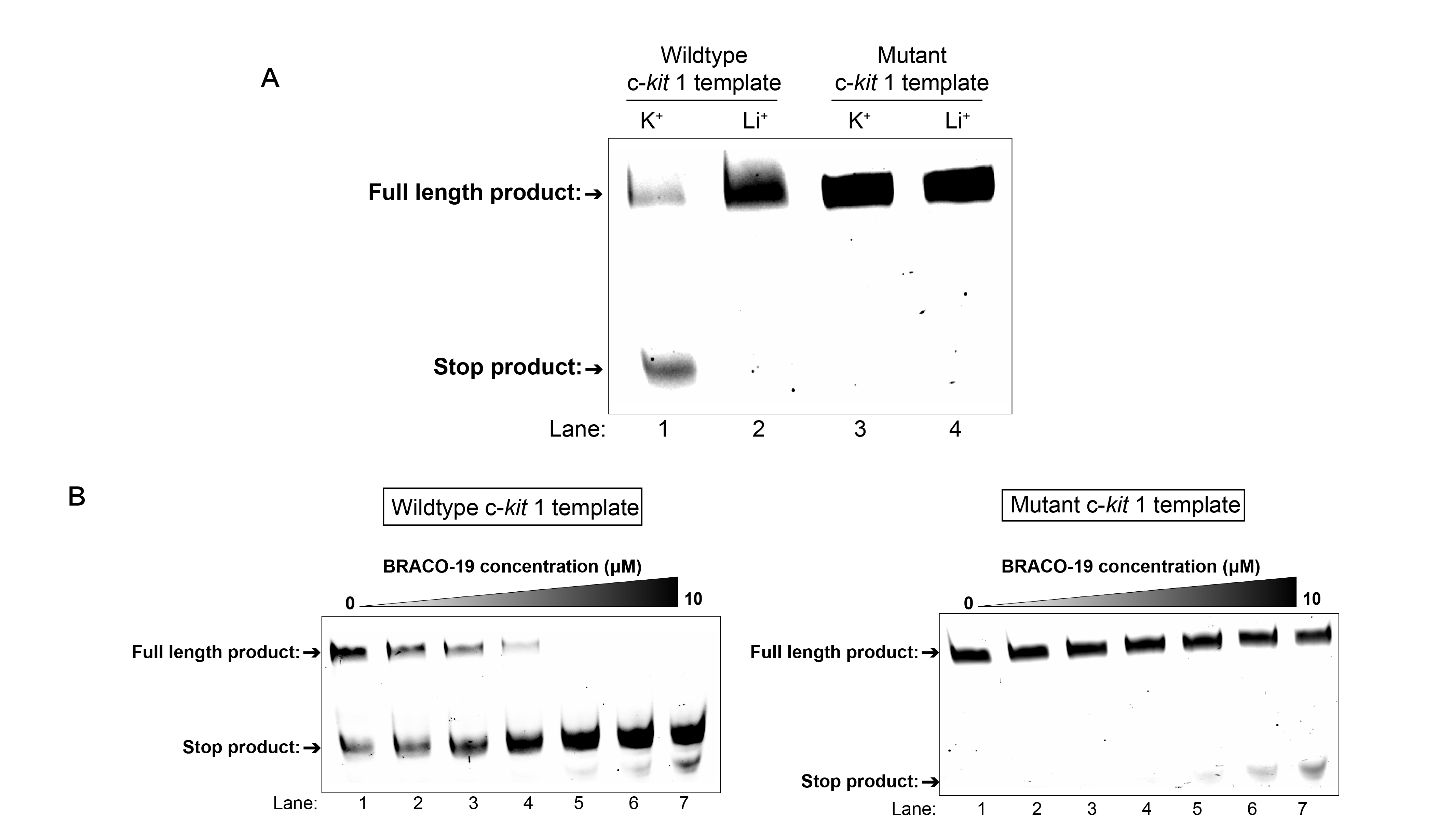
**

**Figure S14.** DNA polymerase stop assay. (A) Primer extension reaction of the wildtype/mutant c-*kit* 1 template under K^+^ and Li^+^ conditions. (B) Denaturing PAGE for the DNA polymerase stop assay in the presence of wild-type or mutant parallel dG4 c-*kit* 1 templates with increasing concentrations of BRACO-19 (lanes 1-7:0, 0.5, 1, 2, 5, 8, and 10 μM).

**
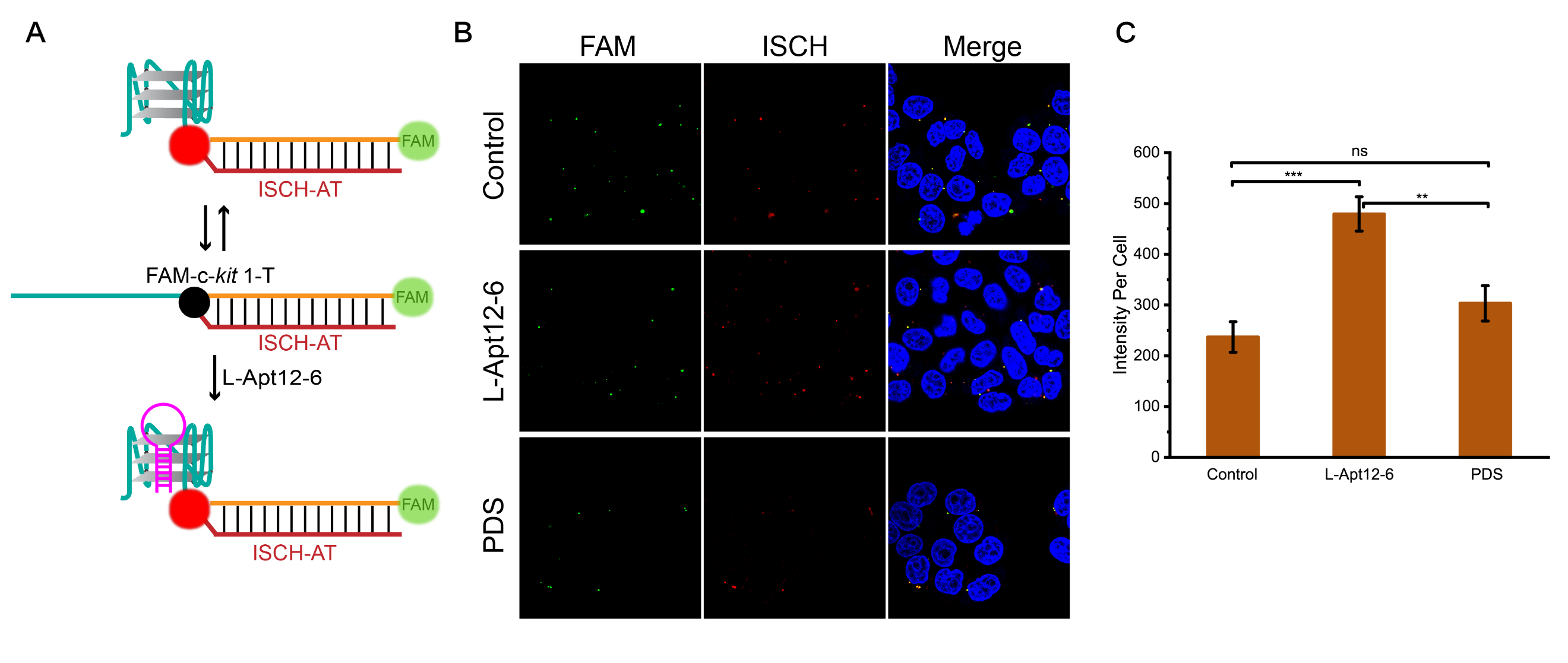
**

**Figure S15.** GTFH probe for c-*kit* 1 dG4 detection. (A) ISCH-AT hybridizes with the tail sequence of FAM-c-*kit* 1-T and recognizes c-*kit* 1 dG4. G4 structure formation can ‘turn on’ ISCH fluorescence. (B) Confocal imaging of FAM-c-*kit* 1-T in HeLa cells. The cells were treated with L-Apt12-6 or PDS and stained with ISCH-AT. (C) Average ISCH fluorescence intensity per cell calculated from B.

**
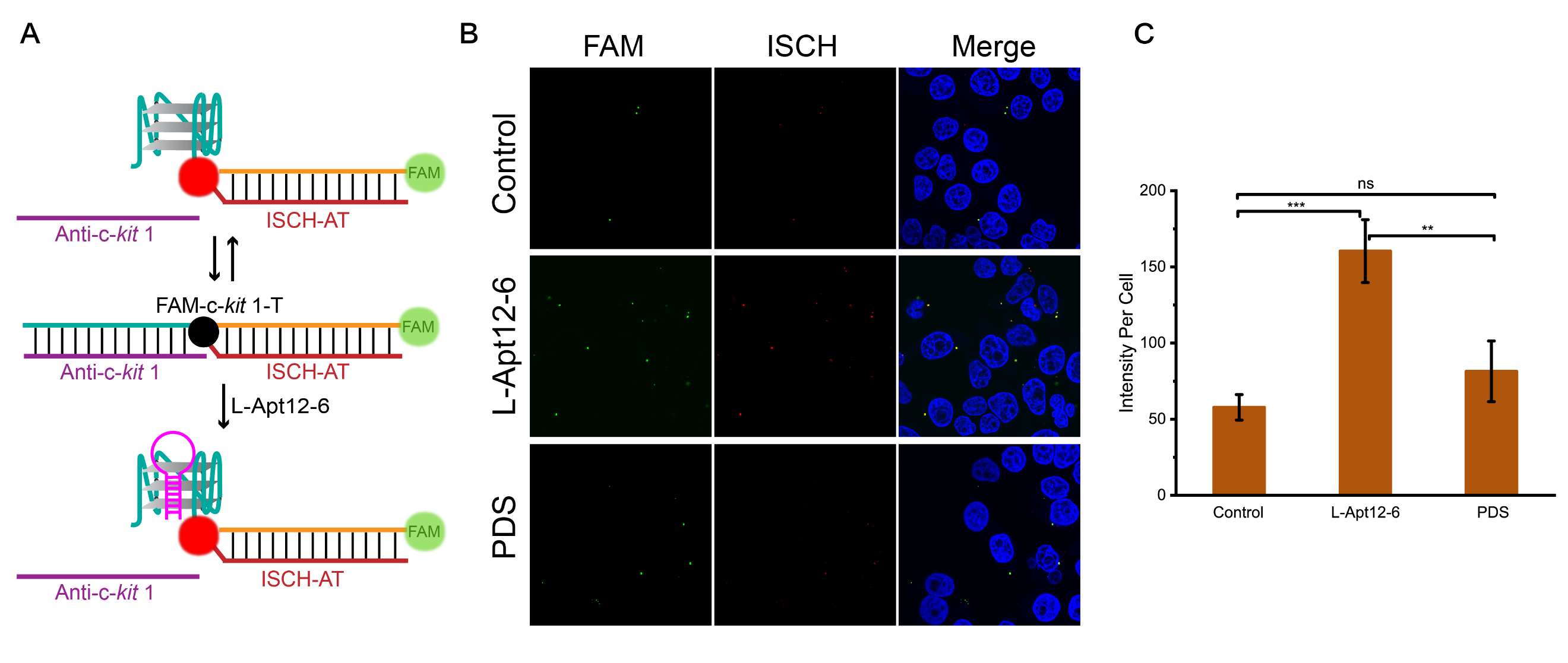
**

**Figure S16.** Detection of duplex-quadruplex transitions in HeLa cells. (A) Anti-c-*kit* 1 hybridizes with c-*kit* 1 dG4. ISCH-AT hybridizes with the tail sequence of FAM-c-*kit* 1-T and recognizes c-*kit* 1 dG4. The formation of G4 structures can induce a duplex-quadruplex transition and ‘turn on’ ISCH fluorescence. (B) Confocal imaging of FAM-c-*kit* 1-T in HeLa cells. The cells were treated with L-Apt12-6 or PDS and stained with ISCH-AT. (C) Average ISCH fluorescence intensity per cell calculated from B.

**
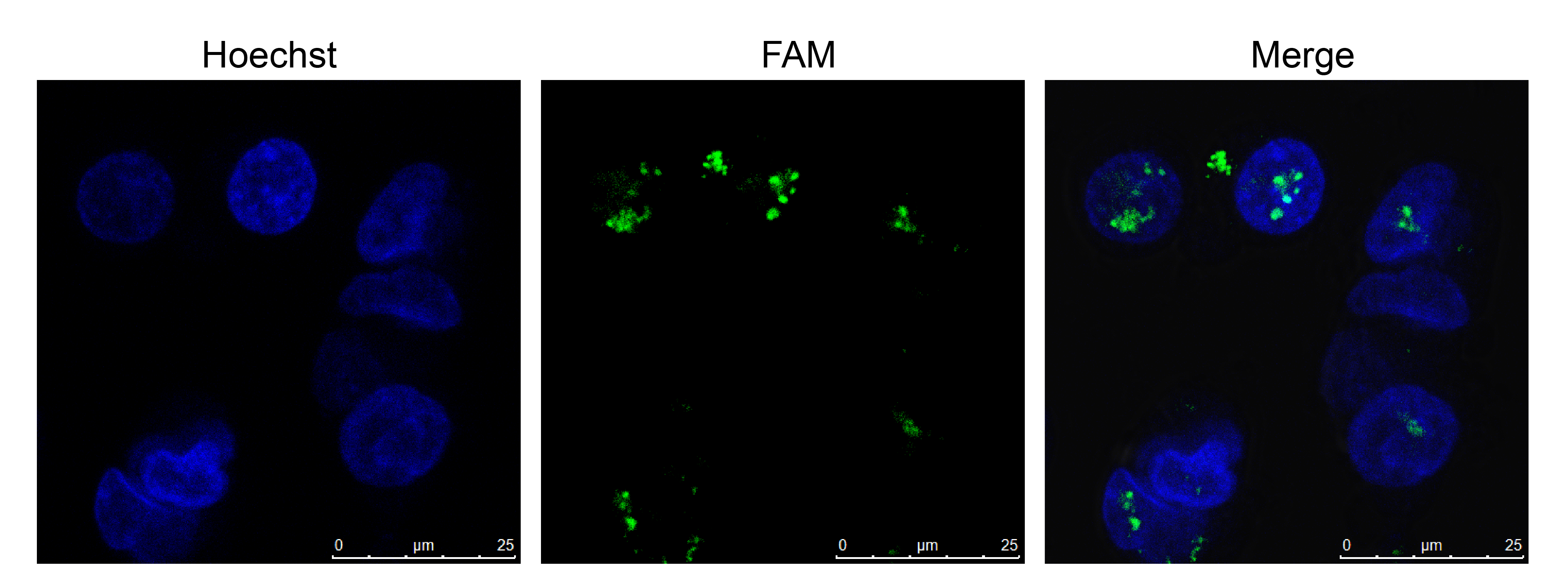
**

**Figure S17.** Imaging FAM-L-Apt12-6 in HEK293T cells. Hoechst 33342 was used to stain cell nuclei.


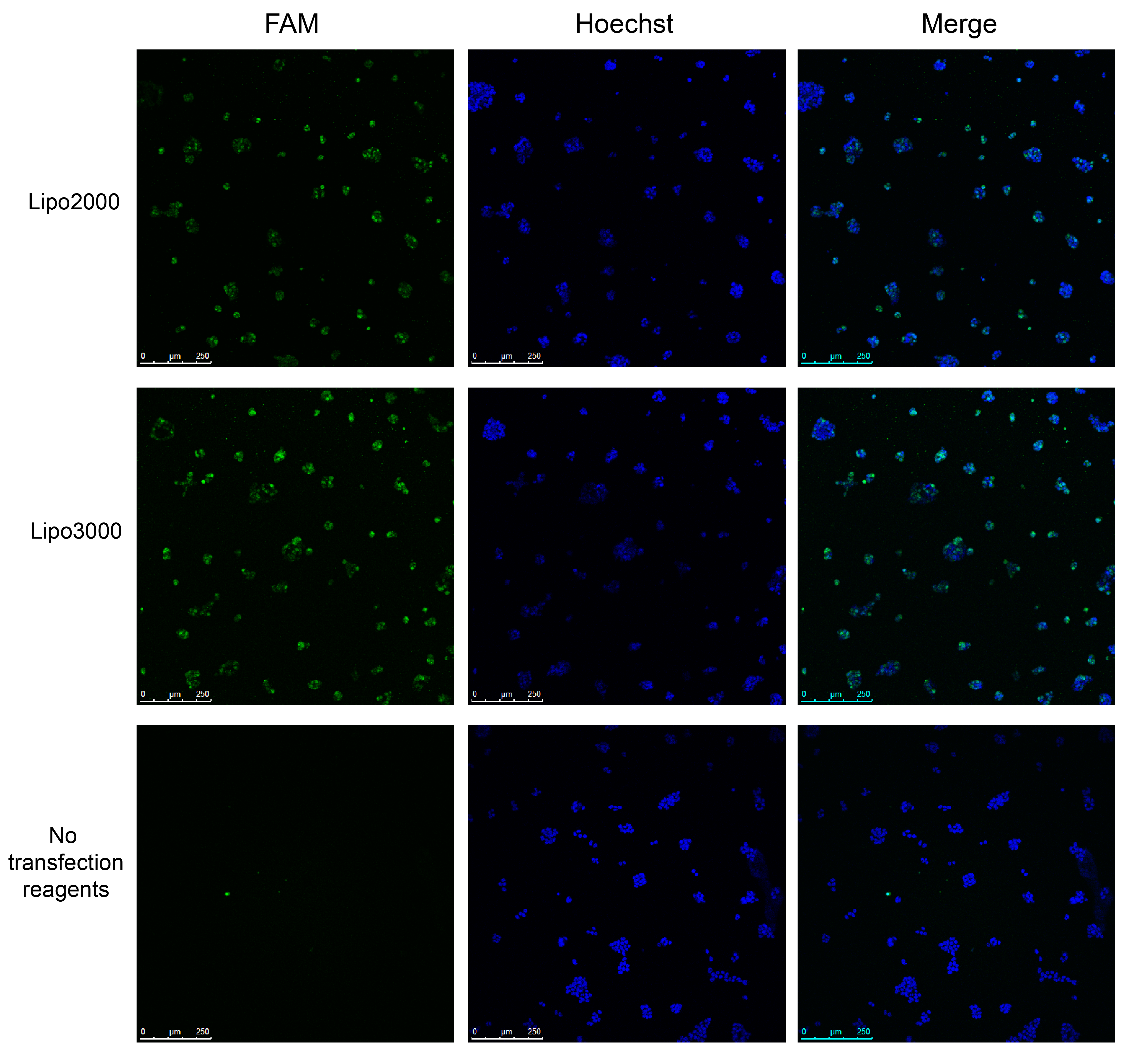


**Figure S18.** FAM-L-Apt12-6 were transfected in HEK293T cells with Lipofectamine 2000 (Lipo2000), Lipofectamine 3000 (Lipo3000) and without transfection reagents. Hoechst 33342 was used to stain cell nuclei.


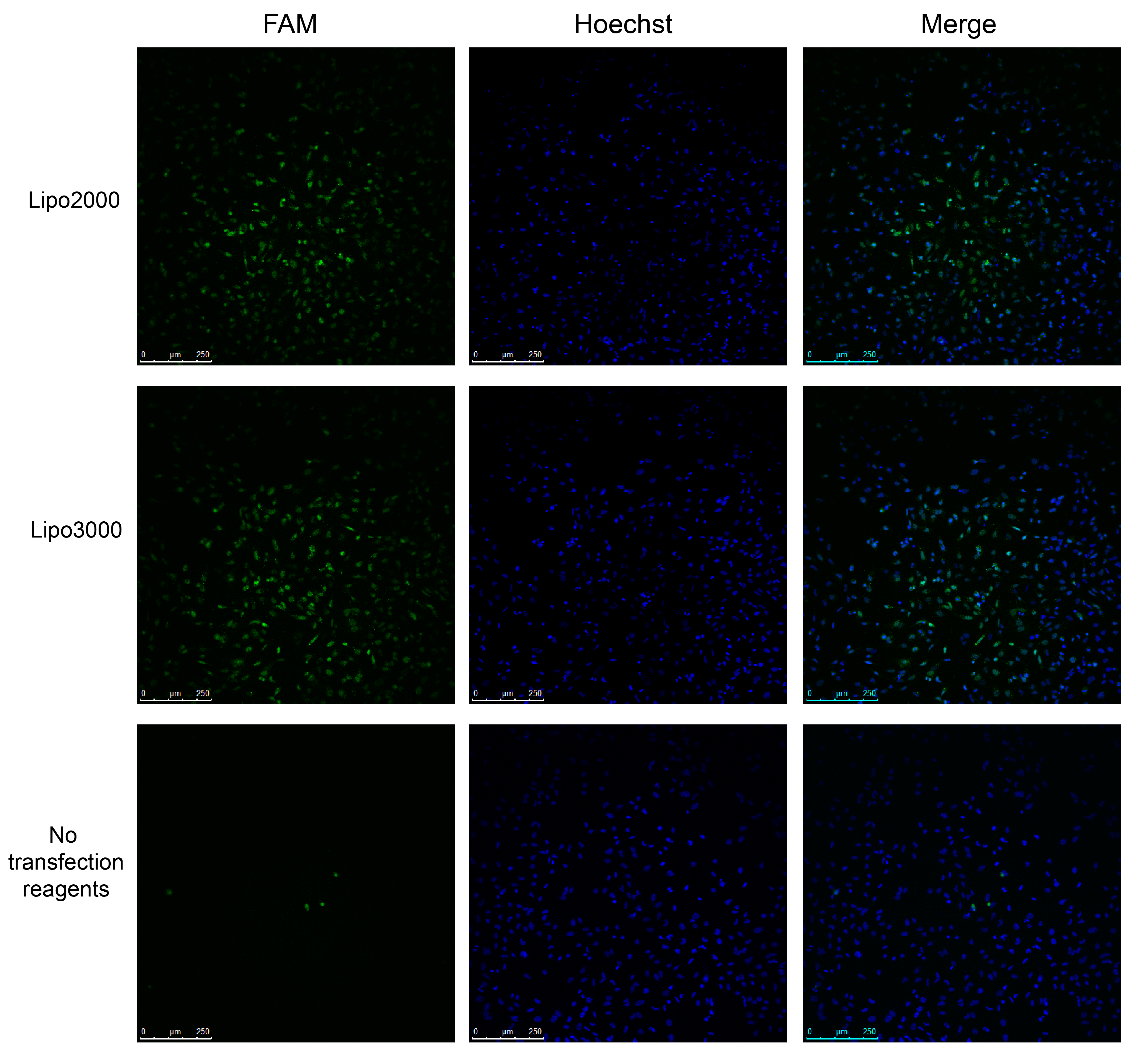


**Figure S19.** FAM-L-Apt12-6 were transfected in HeLa cells with Lipofectamine 2000 (Lipo2000), Lipofectamine 3000 (Lipo3000) and without transfection reagents. Hoechst 33342 was used to stain cell nuclei.


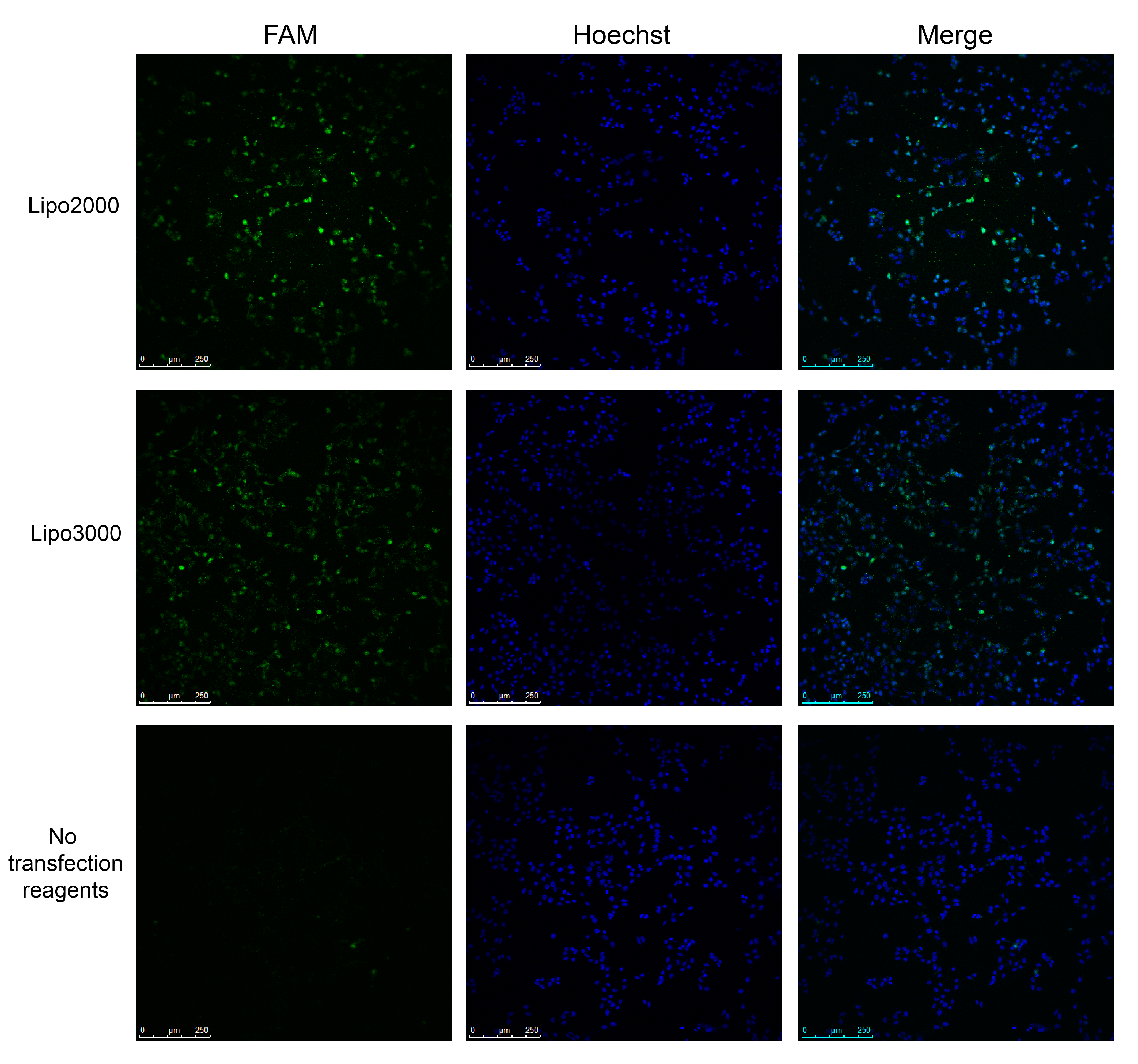


**Figure S20.** FAM-L-Apt12-6 were transfected in HGC-27 cells with Lipofectamine 2000 (Lipo2000), Lipofectamine 3000 (Lipo3000) and without transfection reagents. Hoechst 33342 was used to stain cell nuclei.


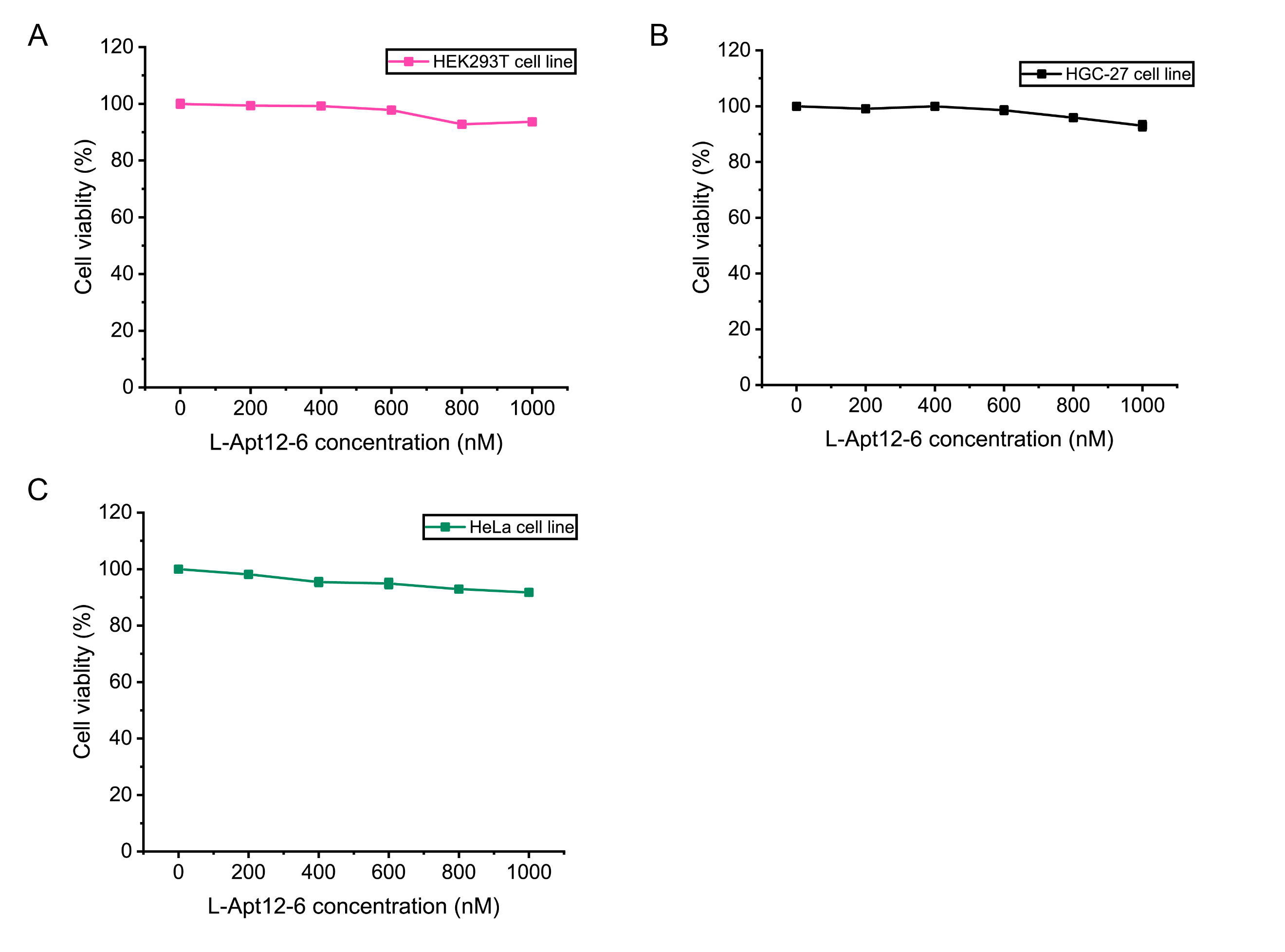


**Figure S21.** Cell Counting Kit-8 (CCK-8) cytotoxicity assays on (A) HEK293T cells, (B) HGC-27 cells and (C) HeLa cells. Cells were incubated with 0-1000 nM of L-Apt12-6 for 48 h, and then were incubated with CCK-8 for 1 h to evaluate cytotoxicity.

**
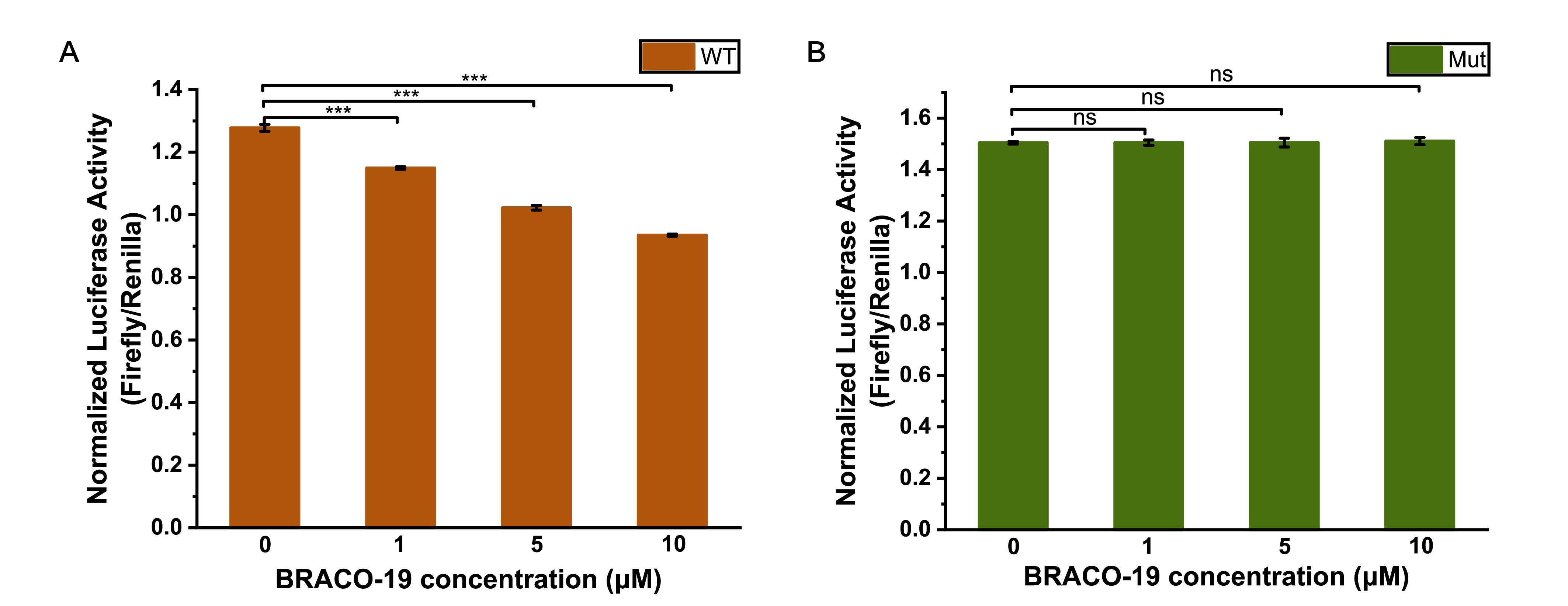
**

**Figure S22.** Reporter gene assay using BRACO-19 treatment. (A) Normalized luciferase activity of cells transfected with the wild-type c-*KIT* plasmid (WT) and BRACO-19. With increasing BRACO-19 (0, 1, 5, and 10 μM) treatment, the luciferase activity level of the WT construct decreased. (B) Normalized luciferase activity of cells transfected with the mutant c-*KIT* plasmid (Mut) and BRACO-19 (0, 1, 5, and 10 μM). However, no significant changes were observed. Error bars represent SEM of three independent replicates. *P<0.05, **P<0.01, ***P<0.001, ns: not significant.


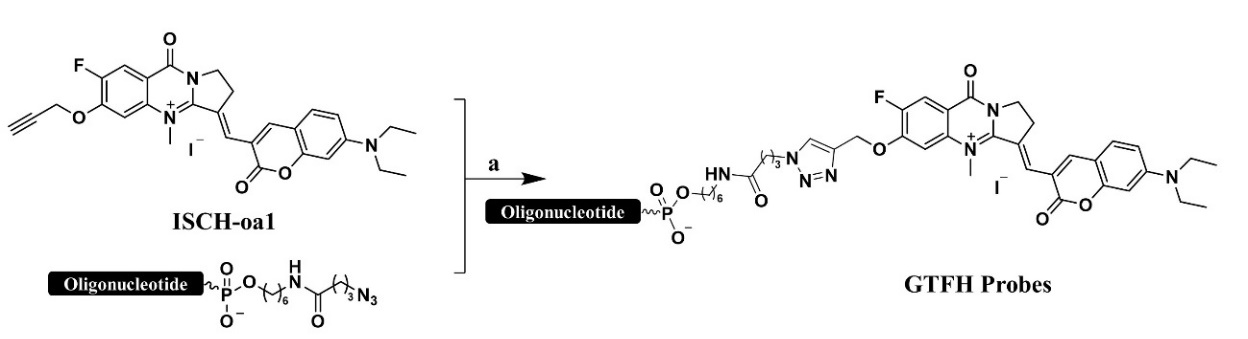


**Figure S23.** The schematic illustration of GTFH probes synthesis using ISCH-oa1 and azido-modified oligonucleotides.
